# Structure and functional impact of glycosaminoglycan modification of HSulf-2 endosulfatase revealed by atomic force microscopy and mass spectrometry

**DOI:** 10.1038/s41598-023-49147-5

**Published:** 2023-12-14

**Authors:** Ilham Seffouh, Mélanie Bilong, Cédric Przybylski, Nesrine El Omrani, Salomé Poyer, Guillaume Lamour, Marie-Jeanne Clément, Rebecca-Joe Boustany, Evelyne Gout, Florence Gonnet, Romain R. Vivès, Régis Daniel

**Affiliations:** 1 Université Paris-Saclay, Univ Evry, CY Cergy Paris Université, CNRS, LAMBE, 91025 Evry-Courcouronnes, France; 2 Université Paris-Saclay, Univ Evry, INSERM, SABNP, 91025 Evry-Courcouronnes, France; 3 Univ. Grenoble Alpes, CNRS, CEA, IBS, Grenoble, France

**Keywords:** Biochemistry, Biophysics, Structural biology

## Abstract

The human sulfatase HSulf-2 is one of only two known endosulfatases that play a decisive role in modulating the binding properties of heparan sulfate proteoglycans on the cell surface and in the extracellular matrix. Recently, HSulf-2 was shown to exhibit an unusual post-translational modification consisting of a sulfated glycosaminoglycan chain. This study describes the structural characterization of this glycosaminoglycan (GAG) and provides new data on its impact on the catalytic properties of HSulf-2. The unrevealed nature of this GAG chain is identified as a chondroitin/dermatan sulfate (CS/DS) mixed chain, as shown by mass spectrometry combined with NMR analysis. It consists primarily of 6-*O* and 4-*O* monosulfated disaccharide units, with a slight predominance of the 4-*O*-sulfation. Using atomic force microscopy, we show that this unique post-translational modification dramatically impacts the enzyme hydrodynamic volume. We identified human hyaluronidase-4 as a secreted hydrolase that can digest HSulf-2 GAG chain. We also showed that HSulf-2 is able to efficiently 6-*O*-desulfate antithrombin III binding pentasaccharide motif, and that this activity was enhanced upon removal of the GAG chain. Finally, we identified five N-glycosylation sites on the protein and showed that, although required, reduced N-glycosylation profiles were sufficient to sustain HSulf-2 integrity.

## Introduction

The human endosulfatases HSulf-1 and 2 (EC 3.1.6.14) are two enzymes in the sulfatase family that are well known due to their catalytic properties and structural features^1–5^. Although HSulf-1/2 were discovered two decades ago^1^, their molecular organization and involvement in physio-pathological processes remain quite elusive^6–9^. Unlike the lysosomal sulfatases, HSulf-1/2 do not participate in the catabolic process. Instead, they catalyze an unprecedented post-biosynthetic modification of the sulfate pattern within the heparan sulfate (HS) chains of cell surface and extra-cellular matrix proteoglycans^3,10,11^. The HSulf enzymes act specifically on the highly sulfated domains of HS, which govern the interaction with various cytokines like growth factors, morphogens, and chemokines^12^. As a result, the cytokine-mediated signals are finely tuned by HSulfs according to the cell needs and in response to some pathological disorders. Being thus at the front line in the signal machinery involving the cell surface HS proteoglycans, HSulfs have been recognized as valuable pharmacological targets in inflammation and tumoral process^7,8,13^. However, the understanding of their biological actions remains unclear due to discrepancies regarding their in vivo involvement and a lack of accurate knowledge of their structural organization.

**Figure 1:**
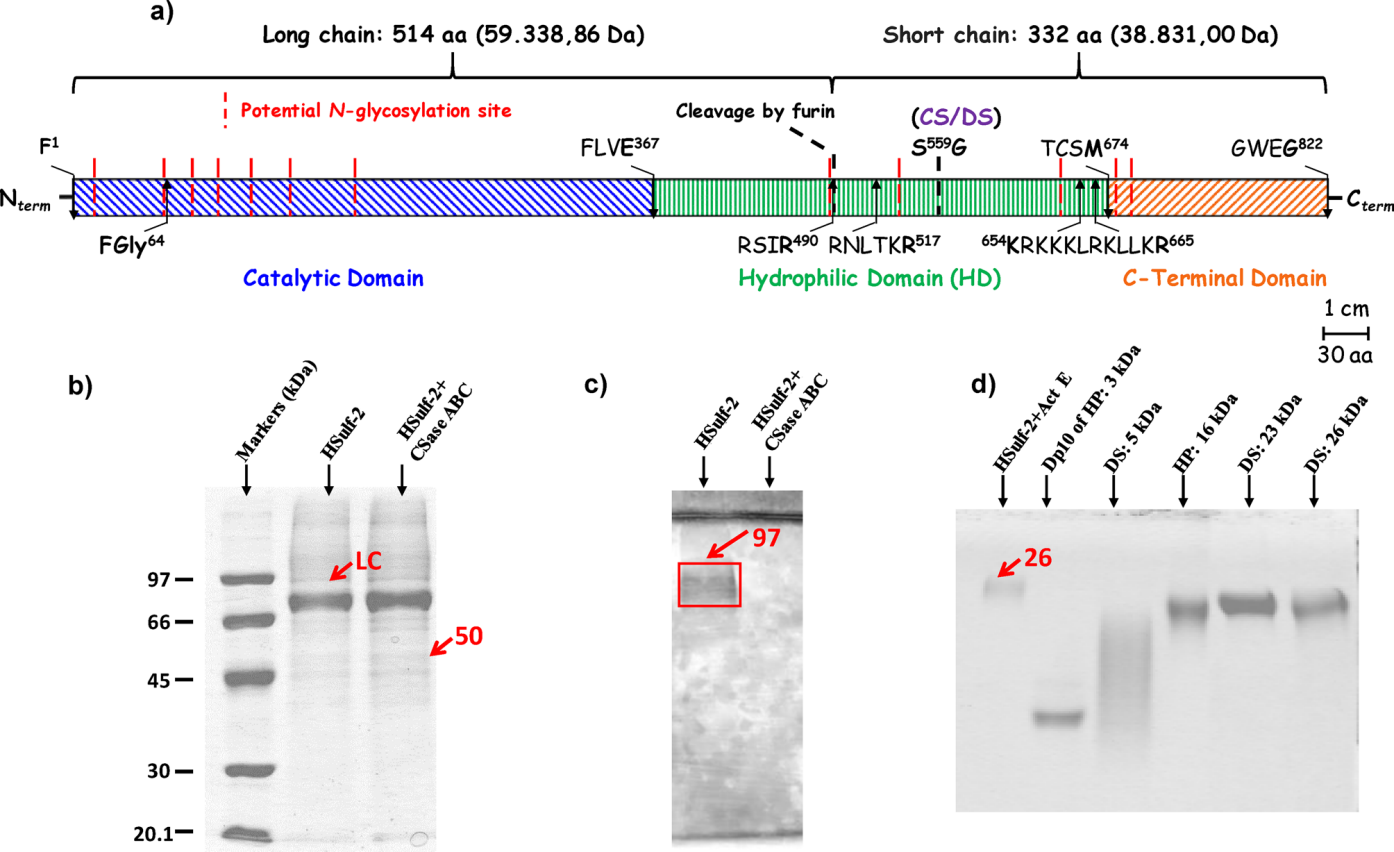
Domain organization of HSulf-2 including a long and a short chain and gel electrophoresis. (**a**) Domains from N- to C- terminus are displayed with their four ending residues: Catalytic Domain (oblique blue lines, 391 aa); Hydrophilic Domain (HD, green vertical lines, 307 aa); C-terminus (oblique orange lines, 148 aa). Red dotted lines: potential N-glycosylation sites. FGly40: formylglycine residue, RSIR^490^ and RNLTKR^517^ main and secondary furin cleavage site, respectively; S^559^G: CS/DS based glycosaminoglycan attachment site; ^654^KRKKKLRKLLKR^665^; RSIR490: HD C-terminus basic residues cluster. For the sake of clarity signal peptide was omitted. (**b**) SDS-PAGE analysis of HSulf-2 on 10% polyacrylamide gel and revealed by Coomassie Blue; (Lane 1) Markers, HSulf-2, before (lane 2) and after (lane 3) hydrolysis by chondroitinase ABC (3 μg HSulf-2/well). *LC* long chain. (**c**) SDS-PAGE analysis of HSulf-2 on 10% polyacrylamide gel and revealed by Alcian blue/silver nitrate; HSulf-2 treated by chondroitinase ABC, before (lane 1) and after (lane 2) hydrolysis by chondroitinase ABC (3 μg HSulf-2/well). (**d**) C-PAGE analysis of the CS/DS linked to HSulf-2. (lane 1) HSulf-2 proteolyzed by Actinase E; (lanes 2 to 6) sulfated polysaccharide markers: 3 kDa heparin decasaccharide, 5 kDa dermatan sulfate, 16 kDa heparin, 23 and 26 kDa dermatan sulfate. 27% polyacrylamide gel, Alcian blue staining, 5 μg HSulf-2/well or 2 μg sulfated polysaccharide markers/well. The full-length gels of (**b**–**d**) are included in Supplementary Information (Figs. S1–S3).

Much effort has been carried out to decipher the catalytic mechanism and structural organization of HSulfs, including its extensive post-translational modifications (PTMs)^2,14–17^. In particular, we previously reported the characterization of HSulf-2 by mass spectrometry, revealing a molecular weight (133,115 Da) much higher than the value expected for the whole enzyme from the naked amino acid backbone (98,170 Da). This difference in molecular weight suggested a significant contribution of glycosylation^14^. Examining the sequence of mature HSulf-2 reveals consensus motifs suggesting twelve potential N-glycosylation sites (Fig. 1a). In addition, an unusual O-glycosylation consisting in a glycosaminoglycan (GAG) chain was recently identified as attached to an SG dipeptide motif, which is the canonical GAG attachment sequence observed in proteoglycans^18^. This rarely observed PTM in an enzyme makes HSulf-2 a new and unique member of the proteoglycan family. The molecular weight of the HSulf-2 variant lacking the SG motif without the GAG chain was reduced to 108,388 g mol^−1^, indicating a significant contribution of the GAG chain to the molecular weight of the HSulf-2 molecule^17^. The GAG linked to HSulf-2 has been proposed to belong to the chondroitin/dermatan sulfate (CS/DS) GAG family based on its enzyme digestion by chondroitinase ABC^18^.

In this study, we carried out the comprehensive survey of HSulf-2 glycosylations, with a particular focus on the structural characterization of its GAG chain, providing its disaccharide composition and revealing its attachment to HSulf-2 through a tetrasaccharide linker. Atomic force microscopy imaging of HSulf-2 and mass spectrometry analysis of the enzyme reaction revealed the impact of this unprecedented PTM on structural and catalytic properties. The data collected unambiguously prove the affiliation of HSulf-2 to the proteoglycan family and emphasize the importance of this GAG chain on the enzyme biological activity.

## Results and discussion

### O-glycosylation of HSulf-2: structural characterization of the HSulf-2-bound CS/DS

The O-glycosylation identified in our previous study^18^ consisted in a GAG chain of chondroitin that had yet to be characterized. Therefore, we undertook its complete characterization here to understand its structural and functional impact better. Recombinant HSulf-2 was overproduced in human HEK293F cells yielding an enzyme carrying PTMs and active on the substrates HS and heparin oligosaccharides^15,16^. The furin cleavage of the single chain HSulf-2 pro-enzyme results in the mature enzyme comprising both a long N-terminal chain hosting the active site and a short C-terminal chain containing a hydrophilic domain involved in the anionic substrate recognition (Fig. 1a). While the long chain was readily detected on SDS PAGE at the apparent molecular weight 75,000, the short chain that was expected to have at a lower molecular weight (38,831 for the naked sequence) was barely or not detected on Coomassie blue stained gel (Fig. 1b), as had been reported in other studies^15,18^. As we hypothesized that PTMs, especially the GAG chain, may likely hinder gel revelation by Coomassie blue, we have chosen the gel staining method using Alcian blue, which detects polyanions and, among others, sulfated GAGs in polyacrylamide gel^19^. When combined with silver nitrate, it allows a sensitive detection. SDS-PAGE of HSulf-2 using this combined staining method revealed an additional band at 97,000. Interestingly, the band at 97,000 was not observed with HSulf-2 treated by chondroitinase ABC (Fig. 1c). Instead, the chondroitinase-treated HSulf-2 sample showed a band at 50,000, which could be stained by Coomassie blue, in addition to the long chain band detected at 75,000 (Fig. 1b). The band at 50,000 was identified as the short chain by in-gel trypsin digestion and nanoLC–ESI–MS/MS analysis of the resulting peptides (Fig. S4).

Together, these results indicate that the short chain of HSulf-2 is associated with a CS/DS GAG and that its apparent molecular weight shifted to 97,000 on gel due to the presence of this GAG. We confirmed that CS/DS GAG prevents detection by Coomassie Blue by testing the same staining of the proteoglycan endocan used as a model for chondroitin-associated protein. Similarly to HSulf-2, endocan was weakly stained by Coomassie Blue and well detected on SDS-PAGE after treatment with chondroitinase ABC (Fig. S5). The CS/DS nature of this new PTM was further confirmed by a western blot experiment using the anti-chondroitin monoclonal antibody CS-56^20^. The immuno-detection of membrane-transferred HSulf-2 revealed an intense, broadband at 100,000–250,000, which was absent in chondroitinase-treated HSulf-2 (Fig. S6). To isolate the associated CS, HSulf-2 was proteolyzed by Actinase E. The breakdown of the protein core yielded a unique carbohydrate component detected by carbohydrate-PAGE analysis (Fig. 1d). The chondroitin nature of this anionic carbohydrate PTM was confirmed by its complete disappearance on C-PAGE gel upon the combined digestion of HSulf-2 by Actinase E and chondroitinase ABC, whereas heparinase I, II and III were ineffective (Fig. S7). The apparent molecular weight of the anionic carbohydrate PTM on C-PAGE gel was estimated to 26,000 based on the migration of GAGs standards. This molecular weight value matched very well with the difference between the experimental mass of HSulf-2 determined by MS and the value calculated from the amino-acid sequence^3,10,11^. Thus, the short chain carries a high-mass carbohydrate PTM that alone accounts for about 25% of the total molecular weight of the protein. Taken together, these results reveal the impact of the CS/DS chain on the separation and detection properties of HSulf-2 and explain the unusual electrophoretic and chromatographic responses of HSulf-2 that we previously reported^14,18^.

Since the structural features of the CS/DS chain have yet to be established, we applied a combination of enzymatic, mass spectrometry, and nuclear magnetic resonance (NMR) techniques to determine the structure of the CS/DS chain. To specify the nature of the CS/DS-based PTM, digestions by different chondroitinases of various substrate specificities were carried out. The chondroitinase AC, which hydrolyzes CS-A (4-*O*-sulfate GalNAc-β1-4-GlcUA) and CS-C (6-*O*-sulfate GalNAc-β-1-4-GlcUA), led to the complete digestion of the CS band on C-PAGE gel. The chondroitinase B, which hydrolyzes CS-B (4-*O*-sulfate GalNAc-β1-4-IdoA, also known as dermatan), was also active on the HSulf-2 associated CS (Fig. S8). Together, these results established that the unusual carbohydrate chain decorating HSulf-2 is a GAG chain combining CS-A/C and CS-B. Interestingly, some lower mass bands but of still significant molecular weight were observed on C-PAGE after digestion by the chondroitinase AC and the chondroitinase B, suggesting alternate blocks of chondroitin A/C and of chondroitin B within the GAG chain.

**Figure 2:**
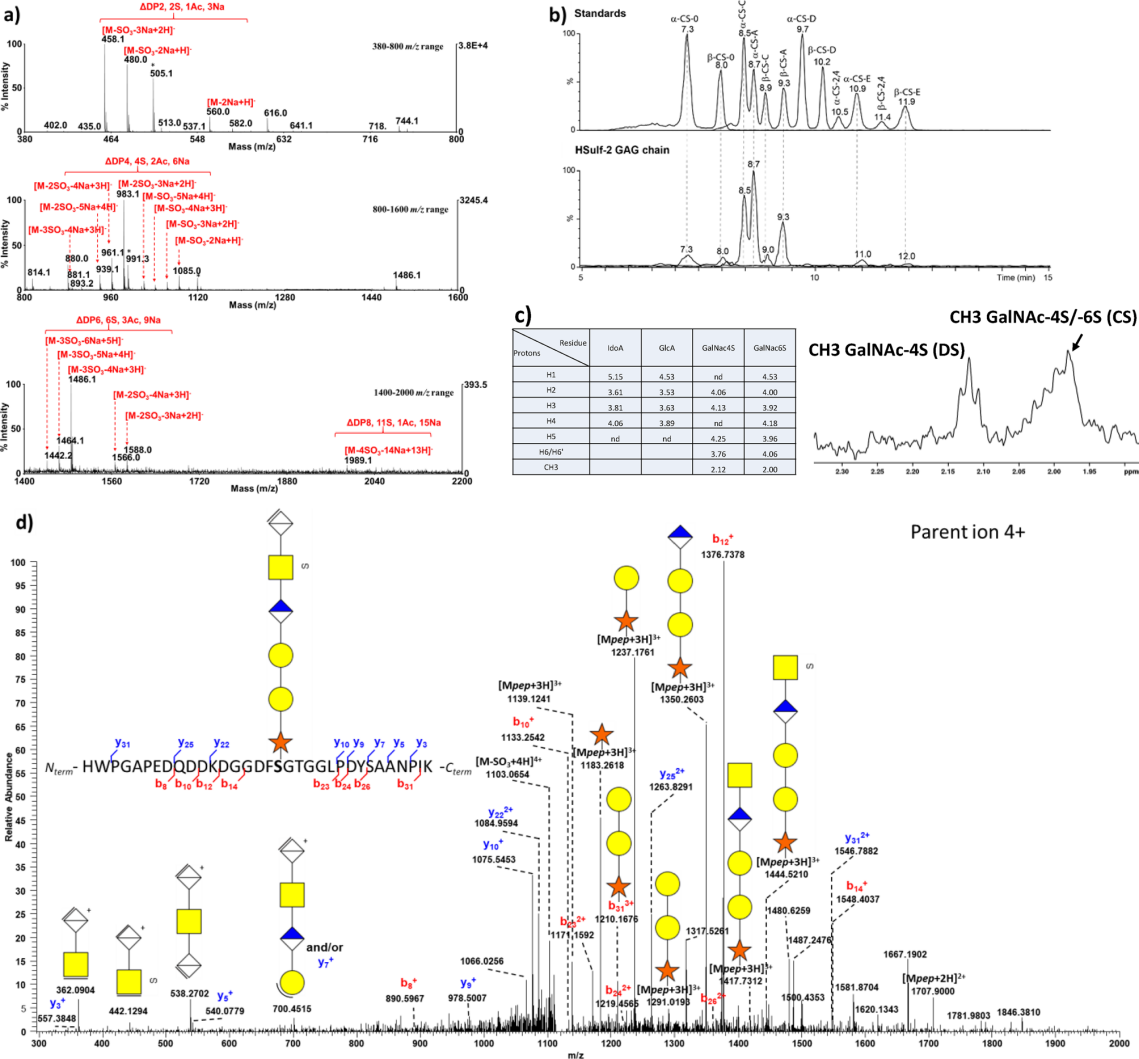
Mass spectrometry and NMR identification of the CS/DS GAG chain attached to HSulf-2. (**a**) Negative reflector MALDI-TOF analysis of CS oligosaccharides from digestion of HSulf-2 by chondroitinase ABC. Mass spectra in the 380–800 *m*/*z* range corresponding to dp2 (top), the 800–1600 *m*/*z* range corresponding to dp4 (middle), and the 1400–2000 *m*/*z* range corresponding to dp6 and dp8 (bottom). M is the molecular ion corresponding to the fully sodiated sulfated oligosaccharide: ΔDP8, 4S, 4Ac, 8Na, M = 2012.13 Da; ΔDP6, 3S, 3Ac, 6Na, M = 1509.10 Da; ΔDP4, 2S, 2Ac, M = 1006.06 Da. (oligosaccharide samples were mixed in a 1:1 volume ratio with the ionic liquid matrix 9 mg/mL HABA/TMG_2_ doped with 100 µM NaCl; * = ions from matrix). (**b**) HILIC-MS of disaccharides released from HSulf-2 by the chondroitinase ABC: Separation of (top) CS disaccharide standards A, C, D, E, and non sulfated CS-0 (EIC of CS-0 in light grey at *m*/*z* 378; EIC of monosulfated CS-C and CS-A at *m*/*z* 458.1 in black; EIC of disulfated CS-D, CS-E and CS-2,4 at *m*/*z* 538.1 in dark grey), and (bottom) disaccharides released from HSulf-2 by the chondroitinases ABC. (**c**) NMR analysis of the CS/DS oligosaccharides released from HSulf-2 by hyaluronidase depolymerization. Proton chemical shifts (ppm)-based identification of sulfated GalNAc, GlcA and IdoA residues from CS/DS oligosaccharides released from HSulf-2 by hyaluronidase (Left table). Methyl group region of the 1D 1H spectrum evidencing the presence of both 4-*O*- and 6-*O*-sulfated GalNAc residues in CS/DS oligosaccharides. (**d**) CID fragmentation spectrum of the glycopeptide ^542^HWPGAPEDQDDKDGGDFSGTGGLPDYSAANPIK^574^ identified by nanoLC–MS/MS analysis as carrying the tetrasaccharide attaching the CS/DS chain to HSulf-2. The selected precursor is *m*/*z* 1122.6935 (4 +). M: mass of the whole glycopeptide; Mpep: mass of naked backbone peptide; dehydrated glucuronic acid (dGlcA): ; *N*-acetylgalactosamine (GalNAc): ; dehydrated N-acetylgalactosamine (dGlcNAc): ; monosulfated-N-acetylgalactosamine (GalNAcS): ; Galactose: ; dehydrated galactose: ; Glucuronic acid (GlcA): ; Xylose:

To further characterize the structure of the CS/DS chain, the oligosaccharides formed by digestion of HSulf-2 by the chondroitinase ABC were analyzed by several MS-based methods. The MALDI-TOF mass spectrum of oligosaccharides isolated after one-hour depolymerization reaction exhibited an ion distribution corresponding to unsaturated UA-GalNAc-based sulfated oligosaccharides ranging from di- to octasaccharides (Fig. 2a). Monosulfated disaccharide was the major depolymerization product, although disulfated disaccharide was also detected (Fig. 2a top). The detected tetra- and hexasaccharides were mainly composed of monosulfated disaccharide units, though disulfated units were also present (Fig. 2a middle). Finally, a low intensity ion at *m/z* 1989.1 (Fig. 2a bottom) was attributed to a tetrasulfated *N*-acetyl octasaccharide (the oligosaccharide sequences for each oligosaccharide are listed in Table S1). These MS data indicate that the CS/DS chain linked to HSulf-2 is mainly made of monosulfated UA-GalNAc disaccharide units.

The major monosulfated constitutive units were identified by hydrophilic interaction liquid chromatography coupled to electrospray mass spectrometry (HILIC-MS) analyses of the disaccharides, which were released by extensive depolymerization using chondroitinase ABC. HILIC-MS allows the separation without prior derivation of unsulfated, mono- and disulfated CS disaccharides, and their on-line identification by negative ionization (Fig. 2b top)^21^. The 4-*O*- and 6-*O*-sulfated ΔUA-GalNAc disaccharides were the two main detected products corresponding to CS-A/B and CS-C, respectively, while only a minor amount of unsulfated and disulfated disaccharides CS-0 and CS-E, respectively, was observed in good agreement with MALDI-TOF MS analysis. The 4-*O*-sulfated isomer dominated the ion signal in mass spectrum with a 40/60 6S/4S signal ratio (Fig. 2b bottom), in agreement with the quantitative determination of the disaccharide composition by RPIP-PLC (Fig. S9). Since the epimerization of GlcA into IdoA requires the prior 4-*O*-sulfation of the GalNAc residue, the observed larger proportion of the 4-*O*-sulfated isomer is a strong indication of the presence of CS-B in the CS chain, consistent with efficient depolymerization by chondroitinase B.

We previously showed that the CS/DS chain of HSulf-2 was susceptible to cleavage by bovine testis hyaluronidases, thereby enhancing the enzyme catalytic activity^18^. To get further physiological insights into this possible regulatory mechanism, we investigated the ability of the human secreted polysaccharidase hyaluronidase-4 to target HSulf-2 CS/DS chain. Hyaluronidase-4 (HYAL-4) is a good candidate since it has been previously shown to be active on CS in vivo^22,23^. The incubation of HSulf-2 with HYAL-4 led to the degradation of the associated CS/DS evidenced by C-PAGE analysis (Fig. S10). This result indicated that the HSulf-2-associated GAG chain is a substrate for this hyaluronidase, which could therefore play an important role in the regulation of the HSulf-2 activity in vivo.

CS/DS oligosaccharides released from HSulf-2 by hyaluronidase depolymerization were analyzed by 1D and 2D NMR (DQF-COSY, TOCSY, ^1^H–^13^C HSQC). NMR analysis of oligosaccharide standards showed that CS-A and CS-C can be differentiated on NMR spectra by distinct chemical shifts depending on the 4-*O* or 6-*O* position of the sulfate on GalNAc, respectively^24^. CS-A and CS-B tetrasaccharides, both 4-*O*-sulfated, can also be differentiated according to their respective glucuronic GlcA and iduronic IdoA epimers, respectively (^1^H–^13^C HSQC spectra, Fig. S11). Due to an important line broadening observed for oligosaccharides issued from the treatment of Hsulf-2 by hyaluronidase, only DQF-COSY and TOCSY spectra could be analyzed reliably. Both spectra showed signals corresponding only to mono sulfated GalNAc residues and to the two epimers IdoA and GlcA, in agreement with the CS chain linked to HSulf-2 (Fig. 2c; Figs. S12, S13). Both GalNAc4S and GalNAc6S were detected, the latter indicating that CS contained CS-C domains (Fig. 2c, Figs. S12, S13). The co-occurrence of IdoA and GlcA residues strongly supports the presence of both CS-A and CS-B within the GAG chain, in agreement with the MS data. The higher intensity of IdoA suggests that CS-B is a significant component of the HSulf-2-bound GAG, consistent with its degradation by chondroitinase B. However, these NMR data do not allow quantitative assessment of the CS/DS ratio. Altogether, MS and NMR analysis data indicate that the CS chain linked to HSulf-2 is predominantly CS-A and CS-B, i.e.*,* with mainly the disaccharide unit GlcA/IdoA-4-*O*-sulfate GalNAc and a smaller proportion of the 6-*O* sulfation.

### The CS/DS GAG chain is attached to HSulf-2 via a tetrasaccharide linker

GAG chains in proteoglycans are linked to the protein core through the tetrasaccharide linker GlcUA-β-(1 → 3)-Gal-β-(1 → 3)-Gal-β-(1 → 4)-Xyl (abbreviated GlcUA-(Gal)_2_-Xyl), which is attached to a serine residue at a conserved SG motif. Two SG motifs are present at Ser559 on the short chain and at Ser484 on the long chain of HSulf-2. We previously showed that HSulf-2 CS/DS chain was found attached to Ser559Gly dipeptide and showed the presence of a tetrasaccharide linker on Ser559 of HSulf-2 found in the conditioned medium of human neuroblastoma SH-SY5Y cells^18^. In order validate these results, we sought out such a tetrasaccharide linker by nanoLC–MS/MS analysis of glycopeptides formed by trypsin proteolysis of chondroitinase-treated HSulf-2 produced in HEK293F cells. Only one glycopeptide detected through the MS/MS detection of the diagnostic ions at *m*/*z* 362.0904, 442.1294, 538.2702 and 700.4515 ascribed to dGalNAc-dGlcA, dGalNAc-dGlcAS, dGlcA-GalNAc-dGlcA, and dGal-GlcA-GalNAc-dGlcA, respectively, was found to have an SG motif. This glycopeptide ^542^HWPGAPEDQDDKDGGDFS*GTGGLPDYSAANPIK^574^ belonged to the short chain and exhibited a mass increment of 1073.2377 at the Ser* residue (Fig. 2d). This mass increment matched very well with the mass of the following monosulfated hexasaccharide GlcUA-GalNAcS -GlcUA-(Gal)2-Xyl, i.e., the tetrasaccharide linker GlcUA-(Gal)_2_-Xyl connected to a remaining chondroitin disaccharide GalNAc-GlcUA. MS/MS data indicate a sulfate group on the disaccharide GalNAc-GlcUA, thus corresponding to a CS unit. Still, we should note that linker sulfation has also been occasionally reported in the literature on the CS/DS biosynthetic pathway^25^. In contrast, linker sulfation has never been observed for HS^26^. These results confirm our initial evidence and establish of the covalent attachment of the CS/DS GAG chain to HSulf-2 via the canonical tetrasaccharide linker present in all proteoglycans^18^. Furthermore, and in line with these previous data, we report the presence of a sulfated GalNAc-GlcUA disaccharide, which is consistent with the extension of the linker by a CS/DS chain during the GAG biosynthesis.

### AFM imaging of HSulf-2

**Figure 3:**
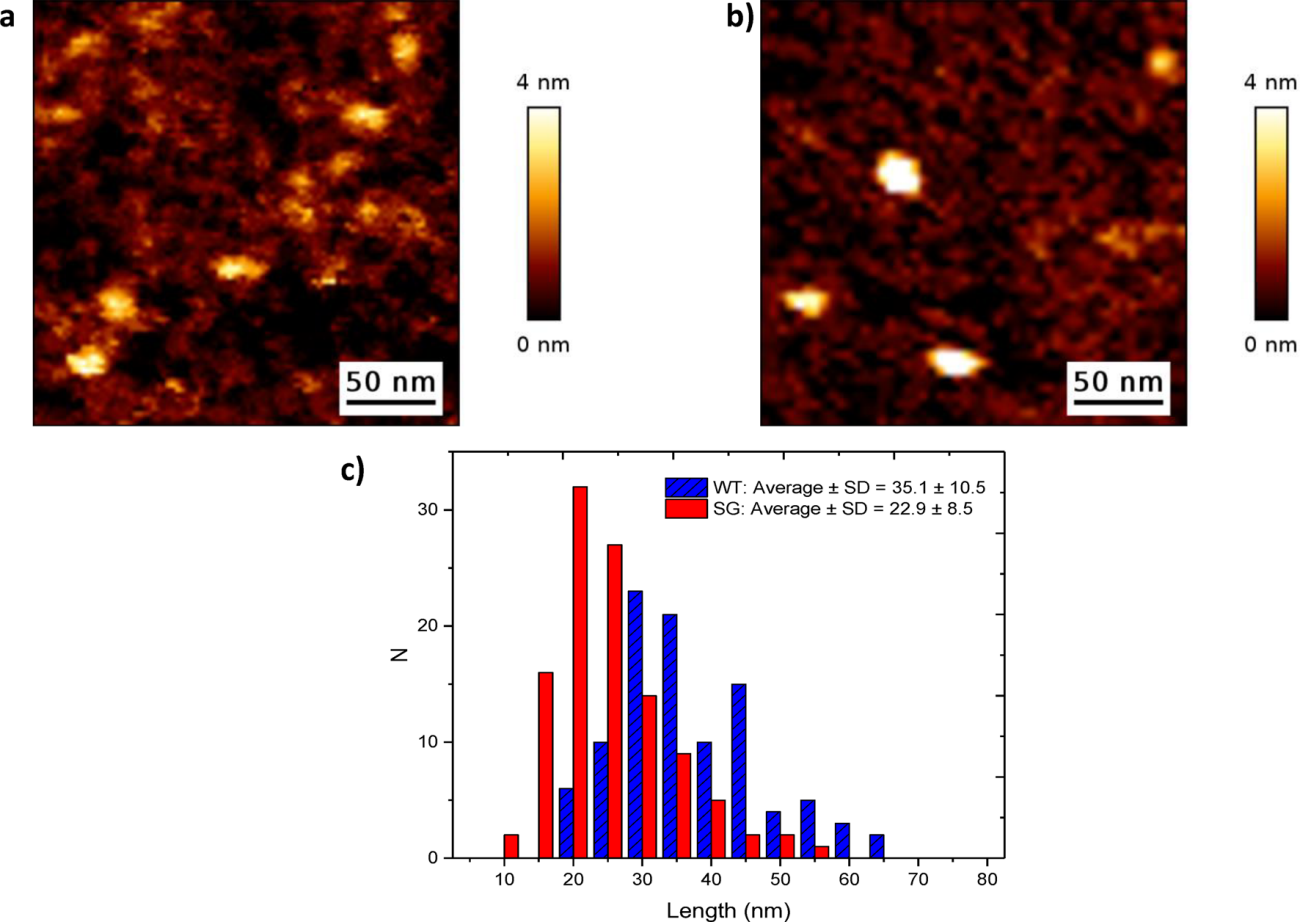
AFM analysis of HSulf-2. AFM imaging of HSulf-2 (**a**) wild type and (**b**) mutant without GAG chain. (**c**) Histograms showing the distributions of the lateral sizes of HSulf-2 wild type and mutant (SG) measured by AFM imaging. N represents the number of counts made on individual molecules.

The chondroitin component associated with the short chain thus represents a major and structurally important PTM of HSulf-2 in addition to the N-glycosylations, accounting for the difference in molecular weight between the whole enzyme (133 kDa) and the naked protein backbone (98 kDa)^3,10,11^. The apparent molecular weight of the short chain in gel and of the whole enzyme in steric exclusion chromatography^15,18^ are both shifted to high values, likely due to the GAG. Furthermore, previous Small angle X-ray scattering (SAXS) of HSulf-2 suggested an elongated molecular shape^18^. Thus, we hypothesized that the GAG chain increases the hydrodynamic volume of HSulf-2 protein, consequently impacting the electrophoretic and chromatographic separation profiles. To better understand the impact of the GAG chain on the size of HSulf-2, we used atomic force microscopy imaging (AFM), which, unlike SAXS, gives access to characterization at the single-molecule scale. After first imaging wild-type HSulf-2 with its GAG chain (Fig. 3a), we then imaged a mutant form of HSulf-2 in which the GAG is absent due to the removal of the SG motif (Fig. 3b). Note that we imaged HSulf-2 molecules in liquid to mimic physiologically relevant conditions, and we used ultra-sharp tips with a low radius of curvature (~ 1 nm) to minimize tip convolution effects. We measured the average lateral sizes of both forms of HSulf-2 and found that the mutant molecule that is deprived of GAG is indeed smaller (average value: 22.9 nm) than the wild-type molecule (average value: 35.1 nm) (Fig. 3c). While GAG accounts for only about 20% of the molecular weight of HSulf-2, its presence induces an increase of about 50% in the size of the endosulfatase as determined by AFM in native (liquid) conditions. With these data, we report for the first time the observation of HSulf-2 hydrodynamic volume by AFM. Results obtained with this alternative approach are fully in agreement with our previous observation that HSulf-2 GAG chain confers a high hydrodynamic radius to the enzyme, as suggested by SEC chromatography and SAXS^18^. In addition, the wild-type HSulf-2 exhibited a broader size distribution, likely resulting from the heterogeneity and polydispersity provided by the GAG chain, in agreement with our previous studies by SEC-MALLS^18^.

### Influence of CS/DS GAG chain on the HSulf-2 sulfatase activity

In addition to its structural impact, our previous study showed that HSulf-2-bound GAG also affected the enzyme activity of the sulfatase. As such, we found that a mutant form of HSulf-2 lacking its GAG chain (HSulf-2ΔSG) exhibited enhanced arylsulfatase activity, using the synthetic substrate 4-MUS, as well as its ability to catalyze the 6-*O*-desulfation of heparin^18^. Here, we sought to explore this further by comparing the ability of HSulf-2 to catalyze the desulfation of 4-MUS following treatment with chondroitinase ABC. As expected, while untreated HSulf-2 exhibited low arylsulfatase activity, the chondroitinase ABC-treated enzyme showed significantly increased activity (Fig. S14).

**Figure 4:**
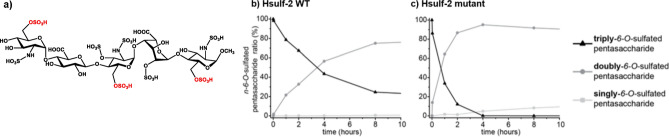
Endosulfatase activity of HSulf-2 measured by HILIC-MS on the heparin pentasaccharide Fondaparinux. (**a**) Structure of the heparin pentasaccharide Fondaparinux. Enzymatic reaction curves obtained from the desulfation of heparin pentasaccharide Fondaparinux using (**b**) HSulf-2 WT and (**c**) HSulf-2 mutant. The curves represent the ratio of the singly- (light grey squares) doubly- (grey circles) and triply-6-*O*-sulfated pentasaccharide (black triangles) in function of time.

Importantly, this increased arylsulfatase activity was similar to that observed for the HSulf-2ΔSG mutant^18^. These results, therefore, confirm, using enzymatic depolymerization, the arylsulfatase modulating activity of HSulf-2 GAG chain. We speculate that HSulf-2-bound GAG may limit the entry of the 4-MUS substrate to the active site and that the presence of a remnant hexasaccharide motif (resulting from chondroitinase digestion) is not sufficient to hinder this access. We then sought to get further insights into the effect of HSulf-2 GAG chain on the enzyme 6-*O*-endosulfatase activity. In our previous report, we used heparin as a substrate. However, the use of this long and highly sulfated polysaccharide may not reflect the actual activity of the enzyme on short, sulfated motifs present within cell-surface HS chains. We, therefore, chose to study HSulf-2 activity on a structurally defined, biologically highly relevant oligosaccharide substrate using the heparin pentasaccharide Fondaparinux. Fondaparinux exhibits three 6-*O*-sulfate groups (Fig. 4), which removal was followed over time by HILIC-MS^21^. An initial 6-*O*-sulfate group was removed by the HSulf-2 enzyme, leading to the concomitant formation of the pentasaccharide with two 6-*O*-sulfate groups and the disappearance of the fully sulfated pentasaccharide (Fig. 4a).

A 50% decrease in fully sulfated pentasaccharide was observed after 3 h of reaction. This is the first time HSulf-2 activity has been demonstrated on a synthetic oligosaccharide of a well-defined structure with the rare 3-*O*-sulfation. Noteworthy, the reaction was notably faster when catalyzed by the HSulf-2ΔSG mutant form lacking the CS GAG, since 50% of the intact pentasaccharide disappeared in less than one hour of reaction (Fig. 4b). The faster turnover by the mutant form is confirmed by the first-order kinetics of consumption of the intact pentasaccharide observed over a duration of 2 h (with 1/k = 1 h, k: reaction rate coefficient) (Fig. 4c). In comparison, the kinetics extended over 8 h with the wild type (with 1/k = 5 h). Unlike HSulf-2 WT, the HSulf-2 mutant form catalyzed the total consumption of the fully 6-*O*-sulfated pentasaccharide, which was achieved within 4 h, while 5% of fully sulfated pentasaccharide still remained after 48 h of reaction with the wild type. This first desulfation was followed by a second one along the oligosaccharide, leading to the formation of the pentasaccharide containing only one 6-*O*-sulfate group. This second desulfation appears earlier and is much more important with the mutant form since it reaches more than 20% after 48 h of reaction against 6% with the wild type. These results provide unambiguous evidence that the antithrombin pentasaccharide-binding motif is a substrate for HSulf-2, and show a clear modulating effect of the GAG on the catalytic properties of HSulf-2 towards a structurally defined oligosaccharide.

### N-glycosylation of HSulf-2

**Figure 5:**
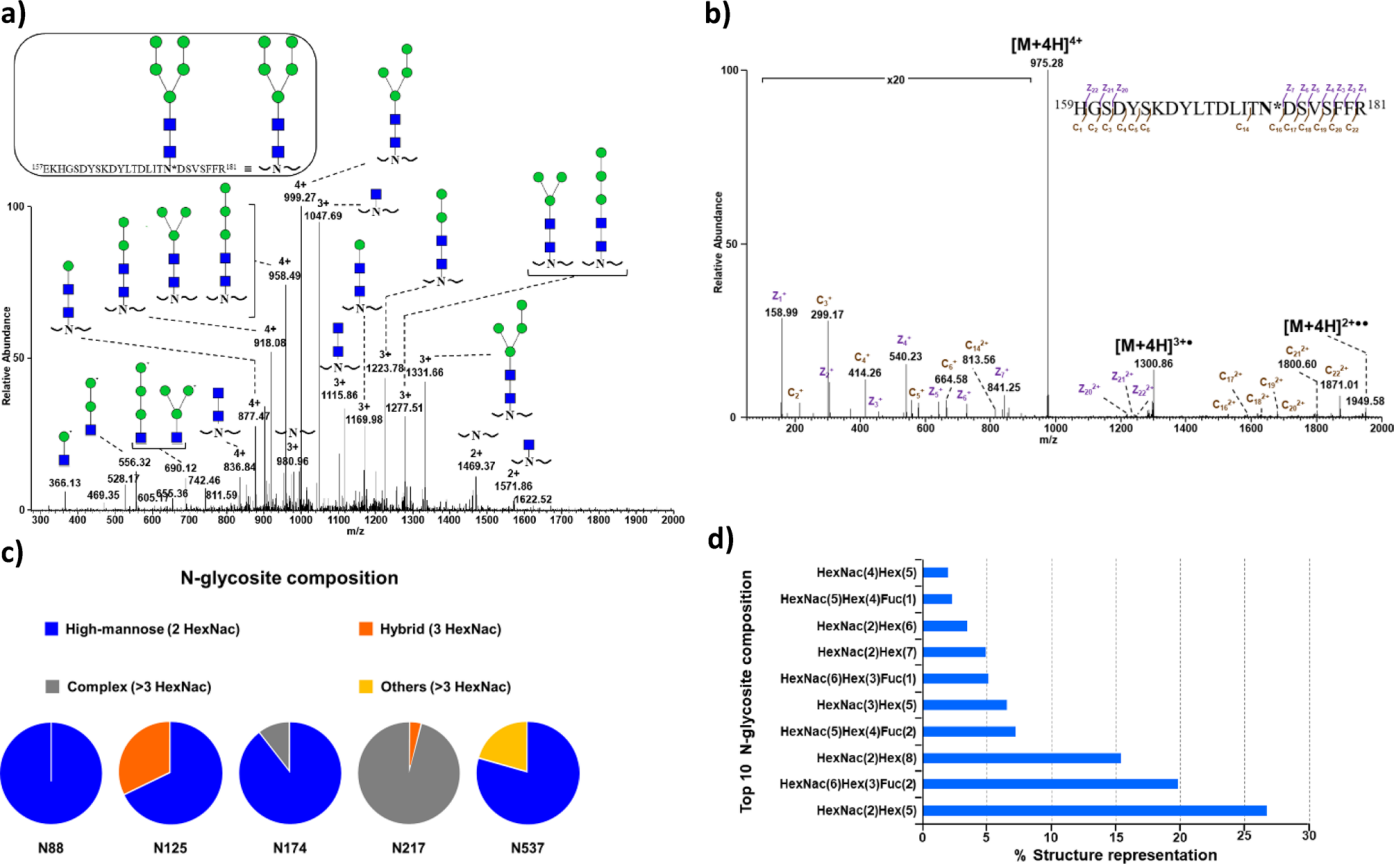
Identification of the main N-glycosites from HSulf-2 by nanoLC–MS/MS analysis. (**a**) CID fragmentation of the glycopeptide ^157^EKHGSDYSKDYLTDLITNDSVSFFR^181^ allowing identification and sequencing of the N-glycosylation at Asn174. The selected precursor in the MS/MS spectrum is *m*/*z* 1039.2092 (4 +). (**b**) ETD fragmentation of ^159^HGSDYSKDYLTDLITNDSVSFFR^181^ allowing sequencing of the peptide backbone. The selected precursor in the MS/MS spectrum is *m/z* 975.4260 (4 +).** M** mass of the whole glycopeptide; N-acetylglucosamine (GlcNAc): ; dehydrated N-acetylglucosamine (dGlcNAc): ; Manose: . (**c**) Distribution of the different types of N-glycans at the five identified sites. (**d**) Structure of the ten most abundant N-glycans identified in HSulf-2.

We previously showed that HSulf-2 is modified by an unexpected O-glycosylation composed of CS without determining its N-glycosylation state^18^. The lower molecular weight gel shift of the long chain observed in SDS-PAGE after treatment by the glycosidase PNGase F indicated the N-glycosylation of HSulf-2^1,17^, in agreement with the twelve potential N-glycosylation sites (motif N-X-S/T, X ≠ P) dispatched over the two chains. We have determined the N-glycans of HSulf-2 by nanoLC–MS/MS analysis of the glycopeptides formed by trypsin proteolysis. The glycopeptides from HSulf-2 were submitted to both CID and ETD fragmentations according to a previously described method^27^. Spectra issued from CID allowed identification of their glycan moieties by detecting the diagnostic oxonium ion HexHexNAc at *m*/*z* 366.13 (Fig. 5a), and ETD established their respective peptide sequence through the dissociation of the peptide backbone (Fig. 5b).

Five N-glycans were identified and unambiguously located within glycopeptides at Asn88, Asn125, Asn174, and Asn217 on the long chain and at Asn537 on the short chain (Table S2) with high-quality spectral evidence. This identification was confirmed by the detection of deamidation introduced by PNGase F on the five Asn glycosylation sites (+ 0.984 mass increment on the MS/MS spectra of deglycosylated peptides, Table S3). The identified main N-glycan structures and their relative abundance were evaluated based on the extracted ion chromatogram of each glycopeptide (Fig. 5c): 52, 7 and 37% of all glycopeptides were ascribed to high-mannose structure, hybrid, and complex glycan (Fig. 5d). The following high-mannose structures were determined: HexNAc(2)Hex(8) at Asn88, and HexNAc(2)Hex(5) at Asn125 and Asn174. As an example, the glycopeptide ^157^EKHGSDYSKDYLTDLITN*DSVSFFR^181^ (two tryptic missed cleavage) comprising the Asn174 glycosylated site was detected at *m*/*z* 1039.21 (monoisotopic mass, 4 +) (Fig. 5a). The corresponding naked peptide backbone was at *m*/*z* 735.10 (monoisotopic mass, 4 +), leading to a net mass difference of 1216.43, which matches very well with the following glycan structure: HexNAc(2)Hex(5) (Table S2). Partial or total loss of mannose residues was also observed on the mass spectrum. The two other N-glycosylation sites, Asn217 and Asn537, exhibited fucosylated complex glycan HexNAc(6)Hex(3)Fuc(2). The N-glycosylation is not responsible for the absence of gel detection of the light chain since it remains invisible in Coomassie blue-stained gel after the removal of N-glycans by enzymatic treatment with PNGase F.

**Figure 6:**
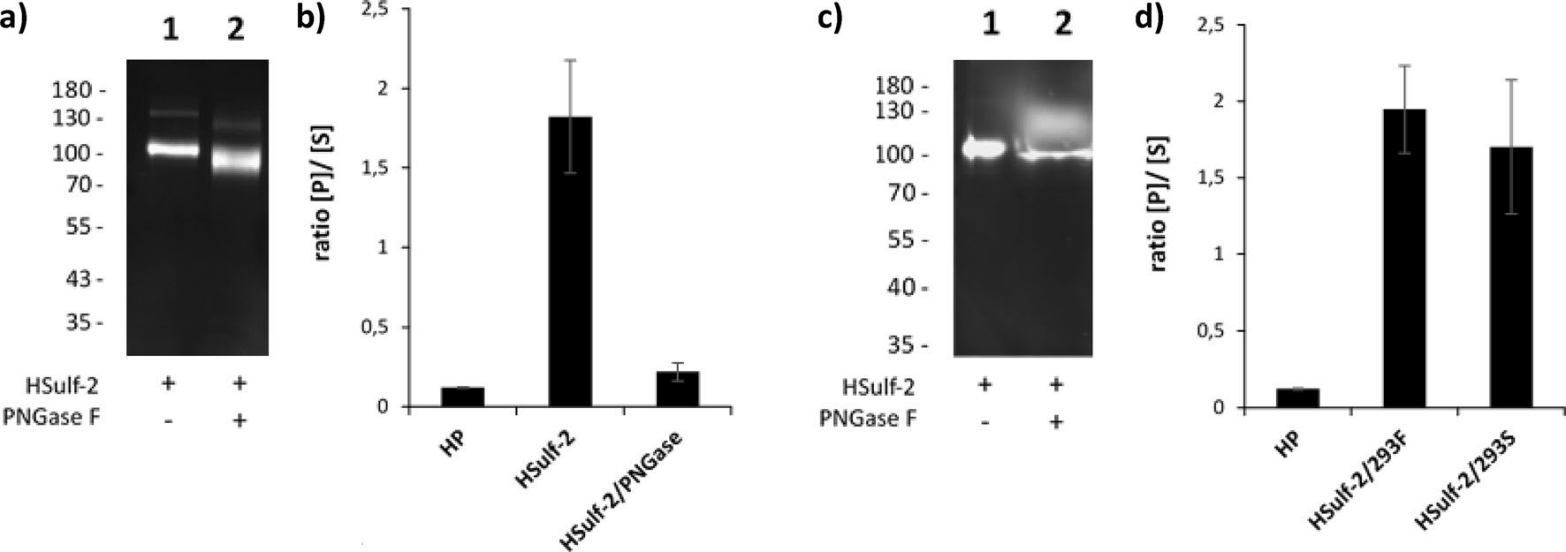
SDS-PAGE analysis and endosulfatase activity of N-deglycosylated HSulf-2. (**a**) SDS-PAGE analysis of SNAP-HSulf-2, treated (lane 2) or not (lane 1) with PNGase F (overnight at 4 °C), detection with the SNAP-Vista Green. Presence of the ~ 23 kDa SNAP Tag allows detection of a single ~ 100 kDa band corresponding to HSulf-2 SNAP tagged N-terminal sub-unit. (**b**) Endosulfatase assay with HSulf-2, or PNGase F treated HSulf-2. Results are expressed as the ratio of detected product/substate, negative control corresponds to untreated heparin (HP), error bars represent SEM of triplicate analysis. (**c**) SDS-PAGE analysis of Hsulf-2 expressed in HEK293F (lane 1, purified protein) or in HEK293S (lane 2, 10× concentrated conditioned medium), detection with the SNAP-Vista Green. (**d**) Endosulfatase assay with purified HSulf-2 expressed in HEK293F or in HEK293S. Results are expressed as the ratio of detected product/substate, negative control corresponds to untreated heparin (HP), error bars represent SEM of triplicate analysis. The full-length gels are included in Supplementary Information (Fig. S15).

Importantly, treatment with PNGase F in non-denaturing conditions (Fig. 6a) resulted in an almost complete loss of the enzyme ability to 6-O-desulfate heparin (Fig. 6b), suggesting that N-glycosylation is important for the enzyme activity and/or structural stability. To investigate further this requirement in N-glycosylation, we expressed HSulf-2 in HEK293S cells, which feature a mutation of Gnt1, a key biosynthesis enzyme catalyzing N-glycan branching. As expected, SDS-PAGE analysis of the protein produced in these cells indicated a slight reduction in molecular weight when compared to its counterpart produced in HEK293F (Fig. 6c). However, the enzyme retained full 6-endosulfatase activity (Fig. 6d), thus suggesting that a low N-glycosylation profile may prove sufficient to sustain enzyme integrity.

## Conclusions

The recent identification of a singular post-translational modification of HSulf-2 by a GAG chain has led to a change of paradigm in the understanding of this elusive enzyme and has paved the way to new perspectives in their structural and functional study^18^. However, as GAG biological properties are strongly associated with their structure and especially sulfation pattern, there was an urgent need to characterize in detail this newly identified GAG chain. In the present study, we used complementary approaches to confirm and provide further details about the CS/DS chain attached to HSulf-2, and we determined the fine molecular features of this polysaccharide. We used C-PAGE to measure the molecular mass of the GAG at 26 kDa, a value which is in agreement with our previous mass spectrometry analysis of HSulf-2^14,18^ and represents a significant percentage (25%) of the global mass of the enzyme. Using AFM imaging, we observed for the first time HSulf-2 at the single-molecule scale, and our data have confirmed that HSulf-2 exhibited an unexpectedly high molecular volume, which may be explained by the size and strong anionic character of the GAG chain, increasing the hydrodynamic volume of the enzyme. We showed that this GAG consists of chondroitin/dermatan sulfate (CS/DS), as determined by MS and NMR analyses and highlighted by its depolymerization by various chondroitin lyases, including human HYAL-4. This is particularly significant as HYAL-4, like HSulf-2, is a hydrolase secreted into the extracellular environment. Hyaluronidase expression is particularly increased in the environment of cancer cells, suggesting a possible interplay between these two enzymes in vivo, to regulate HSulf-2 endosulfatase activity and/or alter the binding of its GAG chain towards extracellular matrix GAG-binding proteins. Using NMR and MS methods, we have analyzed the structural features of the HSulf-2 GAG chain. We demonstrate that this GAG consists primarily of monosulfated disaccharide units, which contain a slight predominance of the 4-*O*-sulfation (61% of ΔUA-GalNac4S). The high proportion of uronic acid under the epimeric form IdoA measured by NMR indicates that DS represents a significant part of the glycosaminoglycan chain, in agreement with its ability to be depolymerized by chondroitinase B. In addition, and in agreement with our previous data, we show that this CS/DS chain is linked by the tetrasaccharide linker GlcUA-(Gal)_2_-Xyl, making HSulf-2 a member of this family and the only one described until now that is enzymatic and secreted without any membrane attachment. The GAG chain is attached to HSulf-2 at the HD domain (Ser559) of the C-terminal chain. This unique hydrophilic domain is only observed on HS endosulfatases and plays an essential role in the interaction with the sulfated polysaccharide substrate. Given these localization and physicochemical features, the GAG chain most likely impacts the interaction and catalytic properties of the enzyme. Indeed, our study shows that the wild-type enzyme with its GAG attached exhibits a slower desulfation rate than the GAG-free enzyme, which may be caused by limited access to the active site or by interference with the interaction of the substrate with the HD domain. Our study is the first to observe and measure HSulf-catalyzed 6-*O*-desulfation directly on an oligosaccharide and not deduce it from a disaccharide composition analysis. In addition, our work clearly indicates that the 3-*O*-sulfation present in the anticoagulant sequences of heparin is not an obstacle to the action of HSulf-2, in agreement with previous results that showed the activity of HSulf-2 on HS enriched in 3-*O*-sulfate^28^.

Finally, this work provides a survey of N-glycosylations present in HSulf-2. A previous study on Quail Sulf-1 reported that these glycosylations were necessary for appropriate cell surface localization and enzyme activity^29^. However, until recently, their functional relevance in the human form had never been investigated^30^. Here, we show that N-deglycosylation upon PNGase F digestion resulted in almost complete loss of enzyme activity, suggesting a role of N-glycans in the enzyme activity or structural organization in agreement with the recently published study^30^. Interestingly, we showed that expression of HSulf-2 in Gnt1^−^ HEK293S cells yielded a fully active protein. This result suggests that a glycosylation motif reduced to the pentasaccharide core is sufficient to sustain HSulf-2 structural and/or functional integrity. Furthermore, this observation raises important perspectives for structural studies that require the production of proteins with homogenous glycosylation profiles. In conclusion, our study provides further insights into the post-translational modifications of HSulf-2 and provides new information about the structural features of its GAG chain, thereby contributing to a better understanding of its functional relevance. Nevertheless, several structural points require further research to clarify further the distribution between CS and DS in the chain and whether this distribution plays a role in the binding of specific partners. Thanks to the possibility of following the enzymatic hydrolysis of 6-*O*-sulfate groups directly on a substrate oligosaccharide, evaluating the influence of various adjacent motifs and the impact of GAG CS/DS on this reaction has the potential to unravel the still elusive catalytic distinction between the two human endosulfatases GAG-lacking HSulf-1 and GAG-bearing HSulf-2.

## Material and methods

### Material

Chondroitin sulfate A (CS-A) from bovine trachea (~ 70%), chondroitin sulfate C (CS-C) from shark cartilage (~ 90%), dermatan sulfate (DS or CS-B) from porcine intestinal mucosa (≥ 90%), heparin pentasaccharide Fondaparinux, 4-methylumbelliferyl sulfate potassium salt (4-MUS), chondroitinase ABC (0.3–3 units/mg), chondroitinase AC (200 units/mg), methanol, acetonitrile, acetic acid, formaldehyde (≥ 36%), Alcian blue 8GX, silver nitrate and Actinase E (P6911) were purchased from Sigma-Aldrich (Saint-Quentin Fallavier, France). Chondroitinase B (1.0 units/mg) was purchased from Iduron (UK). The recombinant human hyaluronidase 4/HYAL4 (6904-GH) was from R&D Systems (Bio-Techne, France). Sequencing-grade modified porcine trypsin (EC 3.4.21.4), was purchased from Promega (Madison, WI, USA). Purified recombinant HSulf-2 was expressed in HEK293F or HEK293S cells and prepared as previously described^15^. Produced HSulf-2 featured N- and C-terminal SNAP and 6His tags that were removed by TEV cleavage during the purification, to yield the protein construct described in Fig. 1. In Fig. 6 only, the non-cleaved protein was used to allow fluorescent detection using a SNAP-tag fluorescent ligand. Rotiphorese 40% acrylamide/bis-acrylamide (29:1) solution was obtained from Roth (Karlsruhe, Germany). Ultra-pure water (18.2 MΩ) was obtained from a Milli-Q purification system (Millipore, France).

### SDS-PAGE and carbohydrate-polyacrylamide gel electrophoresis (C-PAGE)

Gel electrophoresis was performed by using a Mini-PROTEAN^®^ Tetracell system (polyacrylamide gels: 8.3 × 7.3 cm; thickness: 1 mm, Bio-Rad). SDS-PAGE (10% acrylamide) of HSulf-2 samples (3–6 μg), with or without prior treatment by chondroitinase ABC or hyaluronidase, was performed at initial voltage 40 V and then increased by 20 V step every 20 min until 120 V. This voltage was then hold for 90 min. The running buffer was composed of 25 mM Tris, 192 mM glycine, 0.1% SDS, pH 8.3. HSulf-2 samples were loaded in 1× Laemmli buffer in reducing conditions (5× Laemmli buffer: 4 mL 1.5 M Tris–Cl, pH 6.8, 10 mL glycerol, 5 mL β-mercaptoethanol, 5 g SDS and 1 mL 1% bromophenol blue) after heating at 95 °C for 5 min. Molecular weight markers at 97, 66, 45, 30, 20.1, and 14.4 kDa were used (ref. 17-0446-01, Amersham). C-PAGE was performed in 6% acrylamide stacking gel and 27% acrylamide resolving gel, prepared with acrylamide:bis-acrylamide in a 100 mM Tris–HCl buffer, pH 7.8. The C-PAGE separation was performed in the 40 mM Tris, 40 mM acetic acid, pH 7.8 running buffer by applying a constant voltage of 250 V for 45 min. 6 µg of CS/DS samples were loaded into wells with 5 μL of 10% glycerol and 1 μL of 10% phenol red. For sensitive detection, double staining was performed successively using Alcian blue and silver nitrate, as reported elsewhere^19^. The gel was immerged into Alcian blue solution (0.5%) with 2% acetic acid for 10 min under moderate stirring. The Alcian blue staining solution was then withdrawn, and the gel was immersed in water for at least one hour under stirring until revelation of blue-stained bands. The gel was then immersed in fresh silver nitrate solution (0.4% in water) in complete darkness for 10 min. The gel was then rinsed three times with water before adding the developing solution composed of 7 g of sodium carbonate and 80 μL of formaldehyde diluted in 100 mL water. Once new bands appeared, the developing solution was removed and replaced by the 5% acetic acid fixing solution.

### Western blot

10 μg of HSulf-2 was denatured by heating at 95 °C for 5 min in β-mercaptoethanol containing Laemmli buffer, and then submitted to SDS-PAGE. A first staining step by Ponceau red was done to confirm the presence of the protein. Ponceau staining was cleaned in water (3 × 5 min) under stirring. The protein transfer was performed on a nitrocellulose blotting membrane of (0.45 µm, ref 10600003, GE Healthcare) in a cold chamber at 110 V during 40 min in transfer buffer (10% electrophoresis buffer: 25 mM Tris, 192 mM glycine pH 8.3 Bio-Rad 1610771, 20% absolute ethanol, 70% H_2_O). The membrane was rinsed with ultrapure water and blocked by polyvinyl alcohol before the fixation of the anti-CS monoclonal antibody (CS-56, dilution 1:1000 C8035 Sigma-Aldrich) overnight at 4 °C. The membrane was then cleaned three times (5 min each) in Tris-buffered saline with 0.1% Tween (TBS-T, Sigma-Aldrich) before exposition to the secondary antibody anti-mouse IgG (Cell Signaling 7076S) linked to horseradish peroxidase (dilution 1:10,000) for 1 h at room temperature (RT). The membrane was then cleaned three times (5 min each) in TBS-T cleaning solution before incubation with Enhanced chemiluminescent reagent and revelation according to the manufacturer recommendations for the Kit ECL prime western blotting detection reagent (Amsterdam GE Healthcare). SDS-PAGE analysis of HSulf-2 expressed in HEK293S cells or treated with PNGase F was performed likewise, and revealed by fluorescent detection of the SNAP fluorescent ligand SNAP-Vista Green (New England Biolabs).

### GAG depolymerization by chondroitinase ABC

HSulf-2 (3–6 µg) were digested with 5 µL of CSase ABC (10 mU) in 15 µL Tris–HCl 20 mM buffer, pH 7.2, overnight at 37 °C. The resulting CS oligosaccharides were purified using a C18 + Carbon-SPE TopTip cartridge (Glygen, Columbia, MD, USA) as previously described^19^. Briefly, the TopTip cartridge was conditioned twice with 50 µL of 80/20/0.1% (v/v/v) acetonitrile/H_2_O/TFA followed by 3 × 50 µL of water. 50 µL of the digestion mixture was loaded onto a TopTip module, and then the TopTip was washed 5 times with 50 µL of pure water. The oligosaccharides were eluted with 3 × 50 µL 20/80% (v/v) acetonitrile/H_2_O and 50 µL 40/60/0.05% (v/v/v) acetonitrile/H_2_O/TFA. Elution was performed by centrifugation at 3500 rpm for 1 min. Each elution fraction was pooled, freeze-dried, and dissolved in 10 µL of water for subsequent MS analysis.

### GAG depolymerization by hyaluronidase

For NMR analysis, glycerol was withdrawn from HSulf-2 preparation by centrifuge diafiltration in a 3K Centricon unit (15,000 rpm, 5 × 45 min). Digestion of glycerol-free HSulf-2 by hyaluronidase was performed by mixing 30 µL of HSulf-2 (40 µg) and 20 µL of 1000 U/mL hyaluronidase in 70 µL 20 mM sodium phosphate, 77 mM NaCl, pH 7. and overnight incubation at 37 °C. The enzyme reaction was stopped at 95 °C for 5 min. The reaction mixture was freeze-dried in D_2_O. After five freeze-drying cycles, the mixture was resuspended in 60 µL D_2_O to fill a capillary tube for subsequent NMR analysis.

### Combined digestion by trypsin and chondroitinase ABC

Two successive digestions by trypsin and chondroitinase ABC (CSase ABC) were performed to identify tetrasaccharide linker-containing glycopeptides. The first digestion by trypsin was performed as previously described^14^. 2 μL of HSulf-2 sample (3 μg) were diluted in 5.5 μL of 50 mM ammonium bicarbonate (BCAM) pH 8.0 to which 2.5 μL of 8 M urea were added (2 M final), and then incubated for 60 min at RT under moderate stirring. Samples were then reduced by adding 1.1 μL DTT (5 mM final) for 60 min at 37 °C under moderate stirring. Samples were then alkylated by adding 1 μL IAA (20 mM final) and left for 45 min at RT, in the dark. Before performing trypsin digestion, 6.9 μL 50 mM BCAM, pH 8.0, was added to the samples to reach a final concentration of 1 mM urea. The proteolytic digestion was initiated by the addition of 1 μL of 0.4 μg μL^−1^ trypsin and overnight incubation at 37 °C. The trypsin digestion was stopped by heating at 95 °C for 5 min. The digestion by CSase ABC was afterward started by adding 5 µL of 10 mU CSase ABC and 5 µL 20 mM Tris–HCl buffer, pH 7.2, and overnight incubation at 37 °C.

### Proteolysis by Actinase E

3 µg of HSulf-2 were added to 1.2 µL of 0.5 mg/mL Actinase E and 8.8 µL of 20 mM Tris–HCl, pH 7.2 in a final volume of 12 µL. The proteolysis was carried out for 24 h at 37 °C and then stopped by heat at 95 °C for 5 min.

### N-deglycosylation with PNGase F in non-denaturing conditions

HSulf-2 was solubilized in 25 mM Tris, 250 mM NaCl pH 8 and incubated with PNGase F (molar ratio: 1/5) overnight at 4 °C.

### Peptides analysis by reversed-phase NanoLC–MS/MS

Prior to NanoLC–MS/MS analysis, peptides from in-gel digestion of HSulf-2 were resuspended in 20 µL of solvent A (acetonitrile/water/formic acid 2:98:0.1, v/v/v) and submitted to two elution cycles on ZipTip C18 (Millipore) according to the manufacturer procedure aiming to desalt and remove urea. Eluted peptides in 40 µL H_2_O/acetonitrile 20:80, 0.1% TFA, were fully vacuum-dried and stored at − 80 °C until LC–MS/MS analysis. Peptide mixtures were analyzed on a Dual Gradient Ultimate 3000 chromatographic system (Dionex) coupled to an LTQ-Orbitrap™ XL mass spectrometer (Thermo-Fisher Scientific). HSulf-2 peptide digests were resuspended in 30 µL of solvent A, and 5 µL of the peptide solution were injected at 20 µL/min flow rate onto a C18 pre-column (Acclaim PepMap C18, 5 mm length × 300 µm I.D., 5 µm particle size, 100 Å porosity, Dionex). After desalting for 5 min with solvent A, peptide separation was carried out on a C18 capillary column (Acclaim PepMap C18, 15 mm length × 75 µm I.D., 3 µm particle size, 100 Å porosity, Dionex). The gradient started at 100% solvent A for 6 min, ramped to 70% solvent B (water/acetonitrile/formic acid, 20/80/0.1, v/v/v) over 49 min, then to 100% solvent B over 2 min (held 10 min), and finally decreased to 100% solvent A in 3 min. The column was finally re-equilibrated with 100% solvent A for 15 min. The LC eluent was sprayed into the MS instrument with a glass emitter tip (Pico-tip S360-50-15-CE-20-C10.5, New Objective, USA). The LTQ-Orbitrap™ XL mass spectrometer (Thermo-Fisher Scientific) was operated in positive ionization mode. Singly charged species were excluded from fragmentation; dynamic exclusion of already fragmented precursor ions was applied for 300 s, with a repeat count of 1, a repeat duration of 30 s, and a mass exclusion width of ± 1.5 *m*/*z*. The minimum MS signal for triggering MS/MS was set to 500. Microscan was acquired for all scan modes. The Orbitrap cell recorded signals between 250 and 1600 *m*/*z* in profile mode with a resolution set to 60,000 in MS mode. The five most intense ions per scan were dissociated by CID (normalized collision energy 35%, precursor selection window 3 Da). During MS/MS scans, fragmentation and detection occurred in centroid mode in the linear ion trap analyzer. The automatic gain control allowed accumulation of up to 10^6^ ions for FTMS scans and 10^4^ ions for ITMSn scans. Maximum injection time was set to 500 ms for FTMS scans and 100 ms for ITMSn scans. For ETD fragmentation, fluoranthene was used as reagent, the AGC was set to 2.10^5^ ions and reaction time was 50 ms. The analysis of tetrasaccharide linker-containing glycopeptides was made using Byonic v3.6.0 (Protein Metric) software. Searches were performed using the single protein database in the semi-trypsin mode, allowing two missed cleavages. As for the instrumental parameters, the peptide tolerance was set to 10 ppm and the fragment tolerance to 0.6 Da. The search was performed by allowing different modifications on serine residue such as all possible combinations of sulfation and phosphorylation on the tetrasaccharide linker.

### MALDI-TOF mass spectrometry

MALDI-TOF/MS experiments were performed using a Perseptive Biosystems Voyager-DE Pro STR mass spectrometer (Applied Biosystems/MDS Sciex, Foster City, CA) equipped with a nitrogen UV laser (λ = 337 nm) pulsed at a 20 Hz frequency. MS analyses were performed in negative ion reflector mode with an accelerating potential of − 20 kV and a grid percentage equal to 70%. Mass spectra were recorded with the laser intensity set just above the ionization threshold (2500–2800 arbitrary unit) to avoid fragmentation and loss of sulfate, maximize resolution (pulse width, 3 ns), and obtain a maximal analyte signal with minimal matrix interference. The time delay between the laser pulse and ion extraction was set to 250 ns for analytes of dp ≤ 4 or 300 ns for dp ≥ 6. Typically, mass spectra were obtained by an accumulation of 500–1000 laser shots for each analysis and processed using Data Explorer 4.0 software (Applied Biosystems). The ionic liquid matrix (ILM) HABA/TMG_2_ was prepared as previously described^31^. Briefly, HABA was mixed with TMG at a 1:2 molar ratio in methanol, and the obtained solution was then sonicated for 15 min at 40 °C. After removing methanol by centrifugal evaporation in a SpeedVac for 3 h at RT, the ILM was left in vacuum overnight. ILM was then prepared at a concentration of 90 mg/mL. Further dilution to 9 mg/mL in methanol, and the addition of 100 µM aqueous NaCl to prevent excessive desulfation upon the MALDI processing, were achieved extemporaneously to MS analysis. Purified oligosaccharide fractions were mixed with the ILM in a 1:1 volume ratio. Then, 2 µL of the mixture was next deposited on a mirror-polished stainless steel MALDI target and allowed to dry for 5 min at RT and atmospheric pressure. Then, 2 µL of the mixture was next deposited on a mirror-polished stainless steel MALDI target and allowed to dry for 5 min at RT and atmospheric pressure.

### NMR analysis

NMR spectra were acquired at 303 K on a Bruker AVIII HD 600 MHz spectrometer with a triple resonance cryoprobe. Resonance assignments of CS/DS oligosaccharides from HSulf-2 and CS/DS di- and tetra-oligosaccharide standards were performed using 1D, 2D ^1^H–^13^C HSQC, ^1^H–^13^C HMBC, DQF-COSY, and TOCSY experiments. 70 μg of HSulf-2 were added to 200 μL of 20 mM sodium phosphate, 77 mM sodium chloride, pH 7.0 buffer. The resulting HSulf-2 solution was then centrifuged on a Microcon 30 kDa filter unit to remove glycerol from the sample. The filtration was performed five times at 15,000 rpm. 20 μL of hyaluronidase-4 was then added, and the reaction incubated for 48 h at 37 °C. After digestion, the sample was lyophilized five times in D_2_O to remove all traces of H_2_O.

### AFM experiments

AFM experiments were performed with a Nanowizard 4 atomic force microscope (JPK/Bruker) using QI mode and ultrasharp Mikromash tips (radius of curvature of 1 nm, spring constant of 20 N/m, NanoAndMore, France). 20 µL of 50 μg/μL HSulf-2 preparations in 50 mM Tris buffer, 10 mM MgCl_2_, pH 7.5 were deposited on a freshly cleaved mica surface (10 mm diameter, grade V1, NanoAndMore, France). After 5 min, the mica surface was rinsed with deionized water and dried with a nitrogen stream to stop protein adsorption. Then, HSulf-2 adsorbed on mica was rehydrated using a 50 mM Tris buffer, 10 mM MgCl_2_, pH 7.5, and imaged immediately using AFM. The vertical displacement speed of the tip was 25 µm/s, and images were collected at a minimum of 126 pixel × 126 pixel over 600 nm × 600 nm scanning areas. The force setpoint was 3–10 nN, and the reference force height at 1 nN was extracted from the force curves using the Data Processing (DP) software from JPK/Bruker. The lateral size of the molecular species (in the *x*–*y* plane) was determined using the Section Analysis tool of the DP software on the 1 nN-height images.

### Arylsulfatase assay

Fluorescence assay of arylsulfatase activity on 4-MUS was performed with a microplate spectrophotometer (Varioskan LUX multimode reader, Thermo Scientific) and UV-Star^®^ microplates (Grenier, France). HSulf-2 (2 μg) with or without prior digestion with chondroitinases ABC/AC/B or hyaluronidase-4 was incubated at 37 °C with 4 mM 4-MUS in 100 μL 50 mM Tris buffer containing 10 mM MgCl_2_, pH 7.5. Enzyme desulfation of 4-MUS to 4-MU was measured by fluorescence emission at 465 nm (excitation at 360 nm) every 20 s using the “kinetic loop” mode.

### HILIC-MS analysis of CS/DS oligosaccharides

HILIC-MS analysis was performed on a quadrupole time of flight (Q-TOF) mass spectrometer (Xevo G2, Waters) equipped with an ESI source operated in the negative ionization mode. The ion source parameters were optimized as follows to limit the in-source fragmentation of sulfate groups: capillary voltage − 2.5 kV, source temperature 80 °C, and sampling cone voltage 20 V. Experiments were carried out in the resolution mode using the *m*/*z* 50–1200 mass range daily calibrated with a 5 mM ammonium formate solution in H_2_O/MeCN 50:50 and leucine enkephalin as internal standard. HILIC separation was achieved using an Aquity H-class apparatus fitted with a ZIC-cHILIC column (SeQuant^®^ ZIC^®^-cHILIC, 150 × 2.1 mm, 3 μm, 100 Å) as previously reported^21^. Solvent A consisted of ammonium formate at 7.5 mM adjusted at pH 4.0 using formic acid, and solvent B was MeCN. The column oven was set at 20 °C, the autosampler at 10 °C, the flow rate at 300 µL/min and 2 µL of oligosaccharide mixtures were injected for each analysis. The separation of CS disaccharides was carried out as follows: 0 min (24% A), 4 min (24% A), 6 min (35% A), 10 min (35% A), 11 min (85% A), 14 min (85% A), 15 min (10% A), 18 min (10% A), 19 min (24% A) for 6 min of column reconditioning. The raw data were then processed using MassLynx v4.1, and OriginPro 8.5 was used to fit extracted ion chromatogram of kinetic studies.

### Endosulfatase assay

The endosulfatase activity of HSulf-2 assayed on the synthetic heparin pentasaccharide Fondaparinux as substrate was monitored by the above HILIC-MS method with the following modifications of gradient and using H_2_O as solvent B: 0 min (35% A), 15 min (65% A), 16 min (95% A), 18 min (95% A), 19 min (100% B), 22 min (100% B), 23 min (100% A), 27 min (100% A), 30 min (10% A), 35 min (10% A), 36 min (35% A) for 6 min of column reconditioning. The reaction was performed at 37 °C in 50 μL 50 mM Tris buffer, 10 mM MgCl_2_, pH 7.5, containing HSulf-2 (3 μg) and 50 μM of heparin pentasaccharide. The reaction was initiated by the addition of either the HSulf-2 wild type or HSulf-2 lacking the GAG chain in a 100:1 (v/v) heparin pentasaccharide/endosulfatase ratio. Samples of 5 μL of the reaction mixture are taken throughout the reaction course, at 0, 30 min, 1 h, 2 h, 4 h, 8 h, 24 h, and 48 h, and mixed with 15 μL of MeCN. The resulting mixture was then diluted twice in 50:50 H_2_O/MeCN and analyzed by HILIC-MS. The heparin endosulfatase assay on heparin was performed as previously described^15^. Briefly, heparin (25 µg) was digested with HSulf-2 (2 µg) for 24H at 37 °C, then digested with heparinases I, II and III (Grampian enzymes). Resulting disaccharides were resolved and quantified by RPIP-HPLC coupled to post-column fluorescent derivatization, as previously described^32^. Results (3 independent experiments) are expressed as the ratio of the % of product ([ΔHexA(2S)-GlcNS] disaccharide) over substrate ([ΔHexA(2S)-GlcNS(6S)] disaccharide).

### Supplementary Information

Supplementary Information.

## Data Availability

All data generated or analysed during this study are included in this published article [and its supplementary information files]. Raw datasets for NMR and mass spectrometry used and/or analysed during the current study available from the corresponding author on reasonable request.
