# Supplementary material for: Structure and functional impact of glycosaminoglycan modification of HSulf-2 endosulfatase revealed by atomic force microscopy and mass spectrometry
Source: Sci Rep. 2023 Dec 14;13:22263. doi: 10.1038/s41598-023-49147-5 (PMC10721642; doi:10.1038/s41598-023-49147-5)
Supplement: Supplementary file 1 — Supplementary Information. [file 41598_2023_49147_MOESM1_ESM.pdf]

Structure and functional impact of glycosaminoglycan modification of HSulf-2 endosulfatase revealed by atomic force microscopy and mass spectrometry

Ilham Seffouh[1,+], Mélanie Bilong[1,+], Cédric Przybylski[1], Nesrine El Omrani[1], Salomé Poyer[1], Guillaume Lamour[1], Marie-Jeanne Clément[2], Rebecca-Joe Boustany[3], Evelyne Gout[3], Florence Gonnet[1], Romain R. Vivès[2], and Régis Daniel[1,*]

[1]Université Paris-Saclay, Univ Evry, CNRS, LAMBE, 91025, Evry-Courcouronnes, France
[2]Université Paris-Saclay, Univ Evry, INSERM, SABNP, 91025, Evry-Courcouronnes, France
[3]Univ. Grenoble Alpes, CNRS, CEA, IBS, Grenoble, France
[*]regis.daniel@univ-evry.fr
[+]these authors contributed equally to this work

Supporting Information

**Figure S1**	**Page 2**
**Figure S2**	**Page 3**
**Figure S3**	**Page 4**
**Figure S4**	**Page 5**
**Figure S5**	**Page 5**
**Figure S6**	**Page 6**
**Figure S7**	**Page 7**
**Figure S8**	**Page 7**
**Figure S9**	**Page 8**
**Figure S10**	**Page 9**
**Figure S11**	**Page 10**
**Figure S12**	**Page 10**
**Figure S13**	**Page 11**
**Figure S14**	**Page 12**
**Table S1**	**Page 12**
**Table S2**	**Page 14**
**Table S3**	**Page 14**
**Figure S15**	**Page 15**

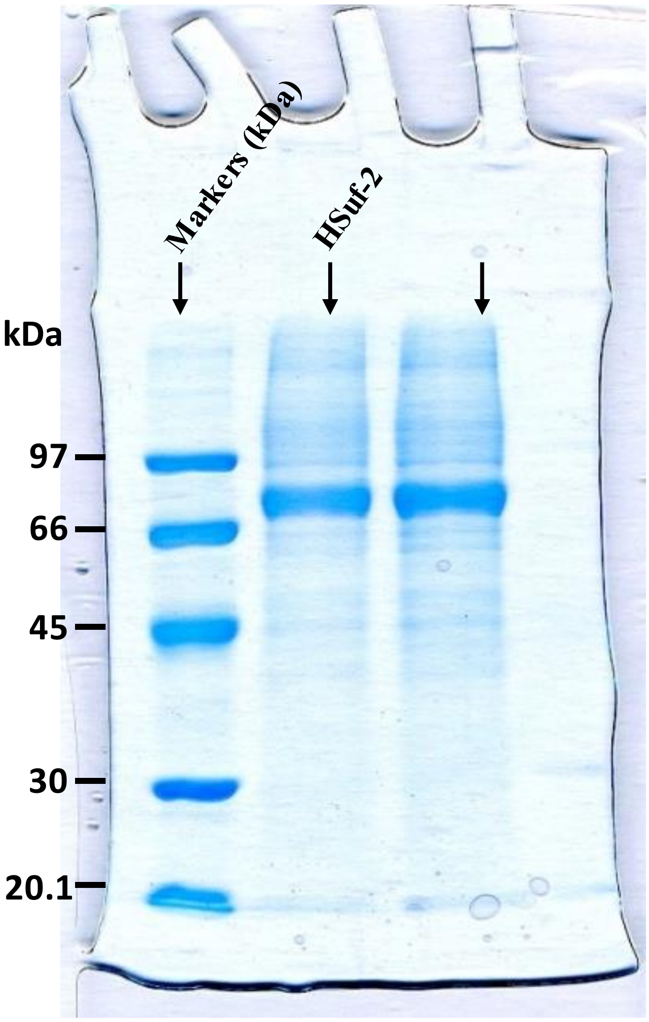

Figure S1. SDS-PAGE analysis of HSulf-2 on 10% polyacrylamide gel and revealed by Coomassie Blue; (Lane 1) Markers, HSulf-2, before (lane 2) and after (lane 3) hydrolysis by chondroitinase ABC (3 µg HSulf-2/well). LC: long chain. c)

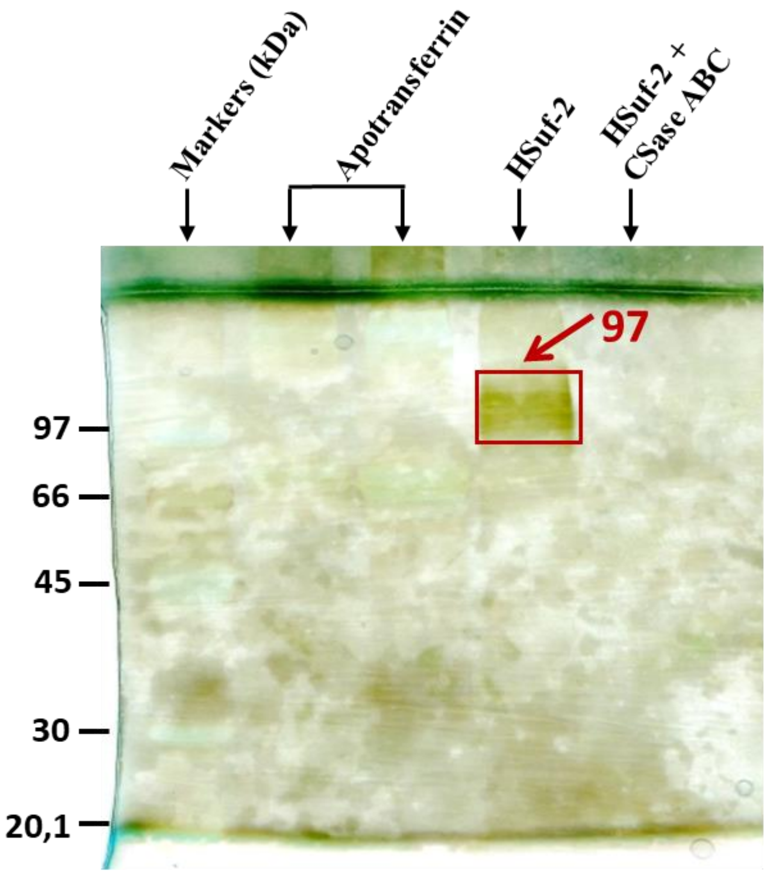

Figure S2. SDS-PAGE analysis of HSulf-2 on 10% polyacrylamide gel and revealed by Alcian blue/ silver nitrate on 10% polyacrylamide gel; (Lane 1) Markers, (Lane 2 to 3) Apotransferrin at 3 and 10 µg as negative control, HSulf-2 treated by chondroitinase ABC, before (lane 4) and after (lane 5) hydrolysis by chondroitinase (3 µg HSulf-2/well).

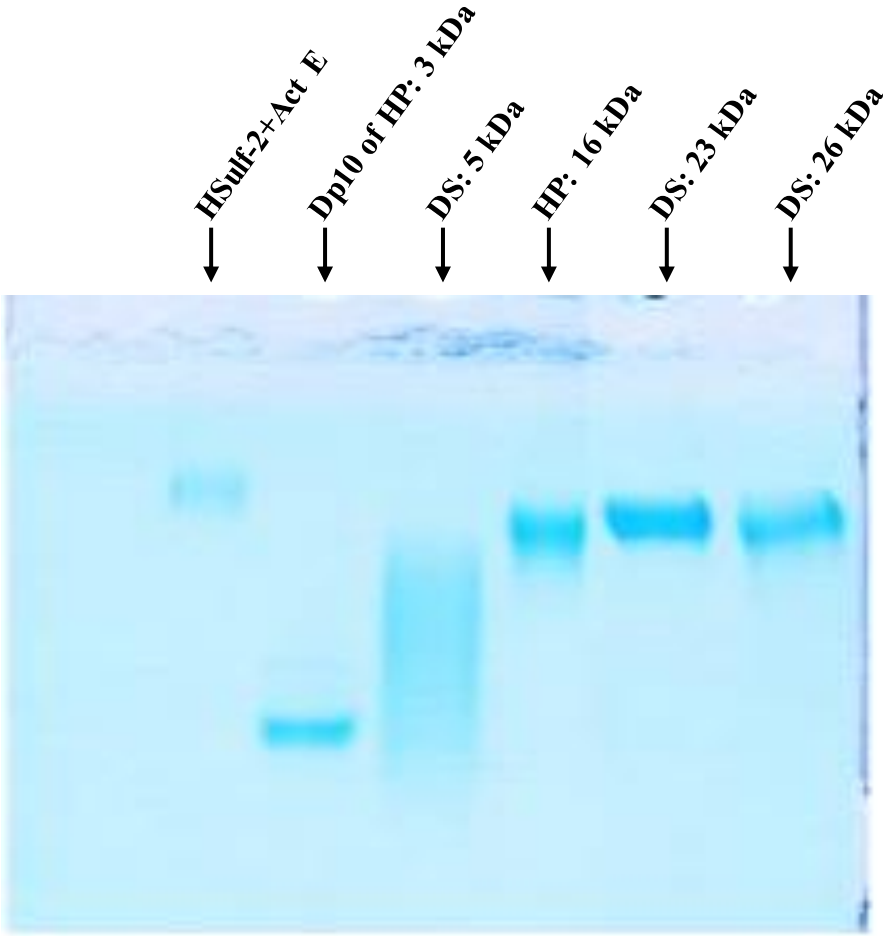

Figure S3. C-PAGE analysis of the CS/DS linked to HSulf-2. (lane 1) HSulf-2 proteolyzed by Actinase E; (lanes 2 to 6) sulfated polysaccharide markers: 3 kDa heparin decasaccharide, 5 kDa dermatan sulfate, 16 kDa heparin, 23 and 26 kDa dermatan sulfate. 27% polyacrylamide gel, Alcian blue staining, 5 µg HSulf-2/well or 2 µg sulfated polysaccharide markers/well.

```
  1 FLSHHRLKGR FQRDRRNIRP NIILVLTDDQ DVELGSMQVM NKTRRIMEQG
 51 GAHFINAFVT TPMCCPSRSS ILTGKYVHNH NTYTNNENCS SPSWQAQHES
101 RTFAVYLNST GYRTAFFGKY LNEYNGSYVP PGWKEWVGLL KNSRFYNYTL
151 CRNGVKEKHG SDYSKDYLTD LITNDSVSFF RTSKKMYPHR PVLMVISHAA
201 PHGPEDSAPQ YSRLFPNASQ HITPSYNYAP NPDKHWIMRY TGPMKPIHME
251 FTNMLQRKRL QTLMSVDDSM ETIYNMLVET GELDNTYIVY TADHGYHIGQ    Long Chain
301 FGLVKGKSMP YEFDIRVPFY VRGPNVEAGC LNPHIVLNID LAPTILDIAG
351 LDIPADMDGK SILKLLDTER PVNRFHLKKK MRVWRDSFLV ERGKLLHKRD
401 NDKVDAQEEN FLPKYQRVKD LCQRAEYQTA CEQLGQKWQC VEDATGKLKL
451 HKCKGPMRLG GSRALSNLVP KYYGQGSEAC TCDSGDYKLS LAGRRKKLFK
501 KKYKASYVRS RSIR
                  SVAIEV DGRVYHVGLG DAAQPRNLTK RHWPGAPEDQ
551 DDKDGGDFSG TGGLPDYSAA NPIKVTHRCY ILENDTVQCD LDLYKSLQAW
601 KDHKLHIDHE IETLQNKIKN LREVRGHLKK KRPEECDCHK ISYHTQHKGR    Short Chain
651 LKHRGSSLHP FRKGLQEKDK VWLLREQKRK KKLRKLLKRL QNNDTCSMPG    47.89%
701 LTCFTHDNQH WQTAPFWTLG PFCACTSANN NTYWCMRTIN ETHNFLFCEF
751 ATGFLEYFDL NTDPYQLMNA VNTLDRDVLN QLHVQLMELR SCKGYKQCNP
801 RTRNMDLGLK DGGSYEQYRQ FQRRKWPEMK RPSSKSLGQL WEGWEG
```

Figure S4. Identification of the band at 50,000 from SDS-PAGE of HSulf-2 treated by chondroitinase ABC**.** Sequence coverage (%) was determined after in gel trypsin treatment and nanoLC-ESI-MS/MS analysis.

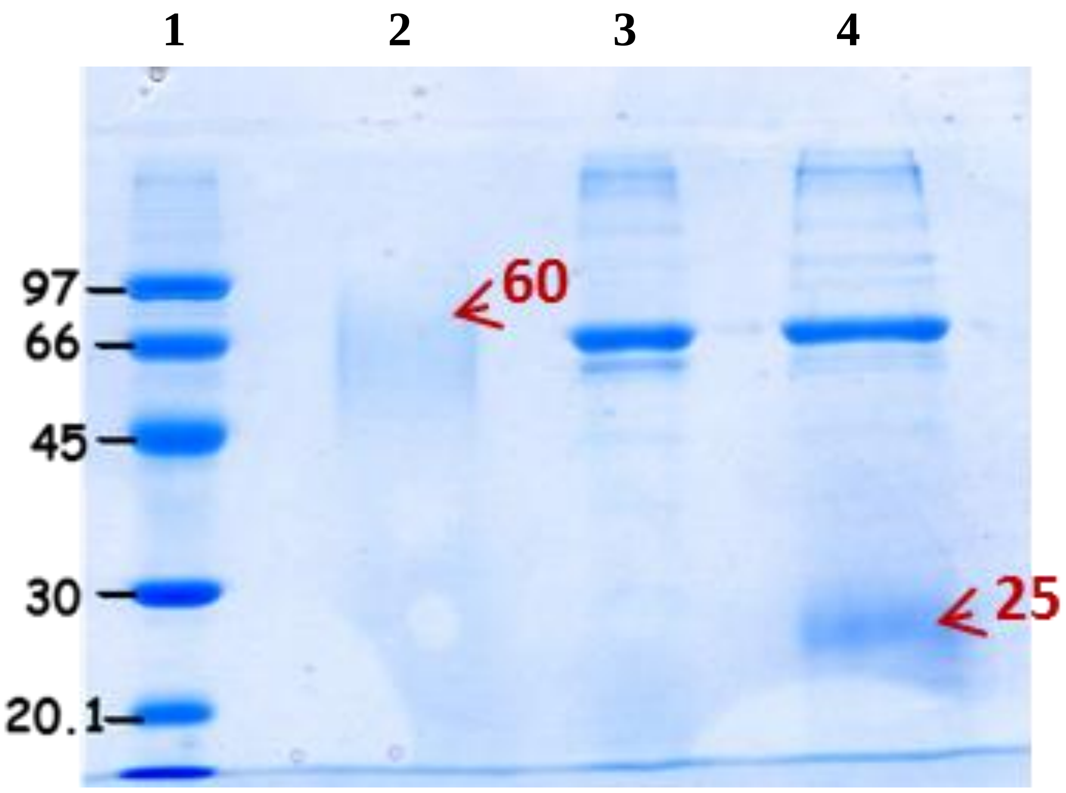

Figure S5. SDS-PAGE analysis of Endocan by Coomassie Blue staining on 12% polyacrylamide gel. Lane 1: molecular weight markers; Lane 2: Endocan (2µg); Lane 3: hondroitinase ABC; Lane4: Endocan after action of the chondroitinase ABC (10 mU) during 24h at 37°C.

a)

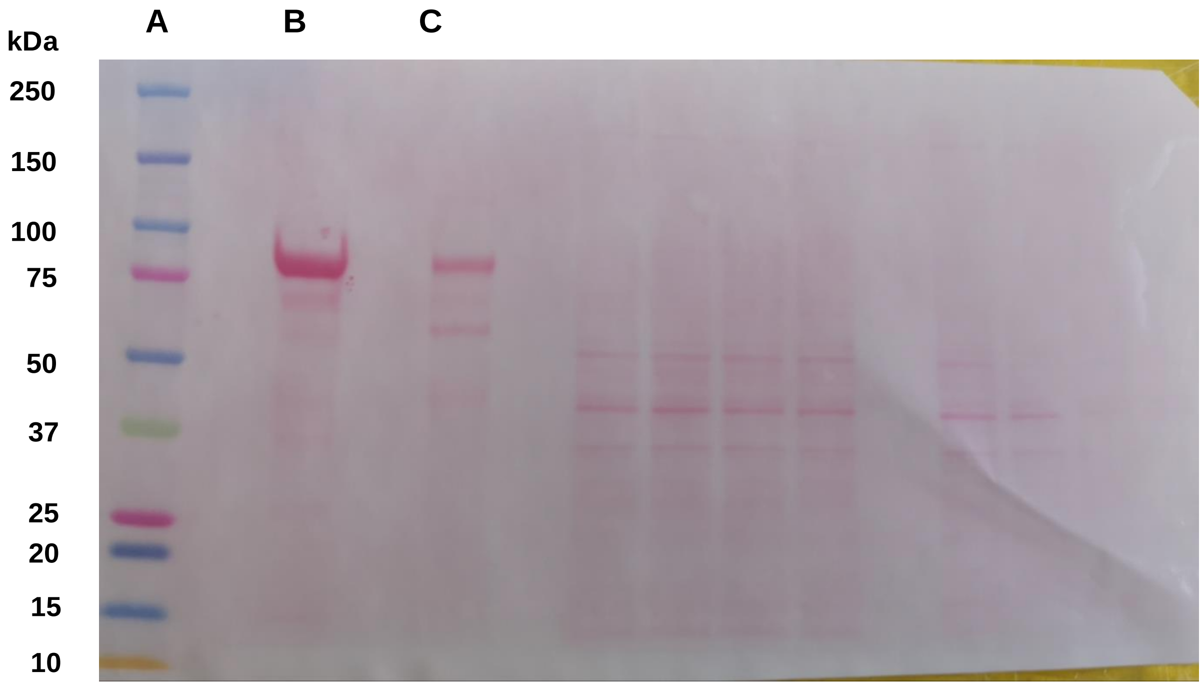

b)

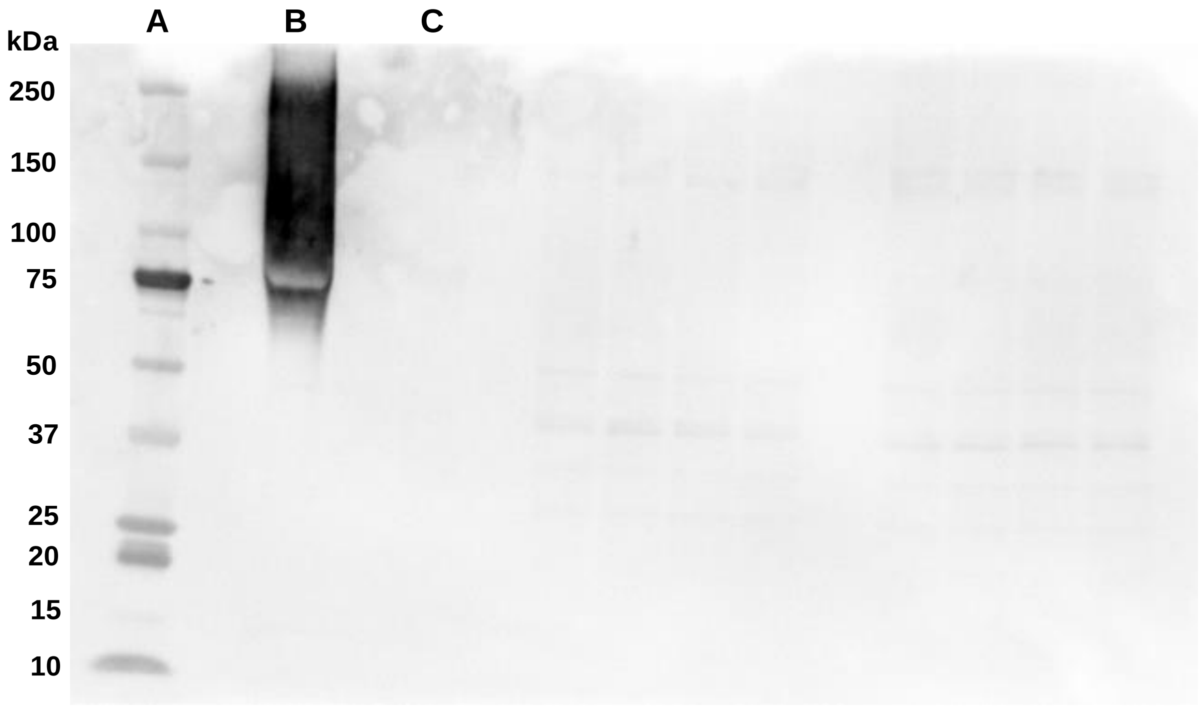

Figure S6. Western Blot of HSulf-2 revealed by anti-chondroitin sulfate antibody. a) Ponceau staining and b) Immunodetection of HSulf-2 by the anti-chondroitin antibody CS-56 before (lane B) and after action of the chondroitinase ABC (lane C). Lane A: molecular weight markers.

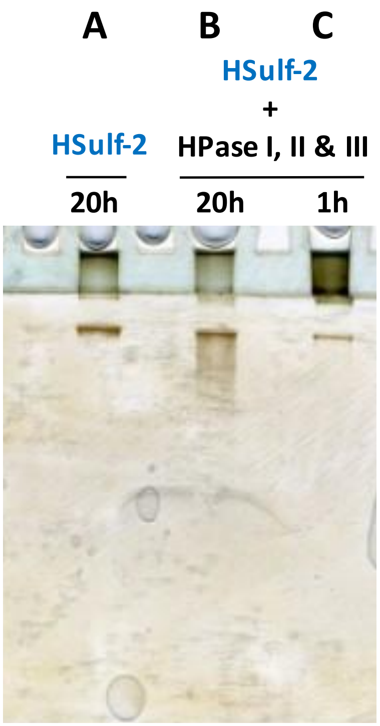

Figure S7. C-PAGE analysis of HSulf-2 with or without treatment by heparinase I, II and III. HSulf-2 without any enzyme treatment incubated at 37°C during 20h (A) or incubated at 37°C with heparinase I, II and III (5mU of each) during 20 (B) and 1h (C). 27% polyacrylamide gel, Alcian blue/silver nitrate staining, 3µg HSulf-2/well.

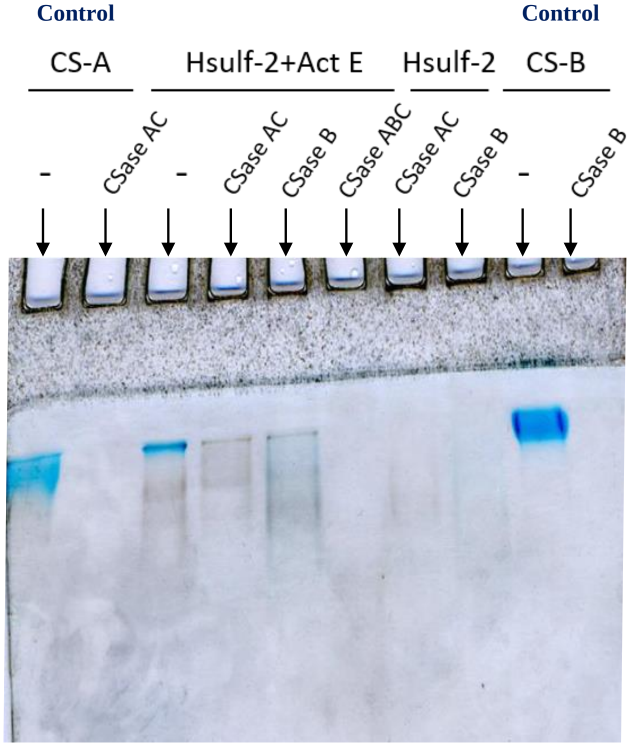

Figure S8. C-PAGE analysis of the depolymerization of the CS/DS linked to HSulf-2 by chondroitinases. HSulf-2 proteolyzed by Actinase E without chondroitinase (4), with CSase AC (5), and with CSase B (6); non-proteolyzed HSulf-2 with CSase ABC (7), with CSase AC (8), and with CSase B. Controls: CS-A (1), CS-A with CSase AC (2), CS-B (9), CS-B with CSase B (10). 27% polyacrylamide gel, Alcian blue/silver nitrate staining, 3µg HSulf-2/well, 2 µg CS-A, -B/well.

a)

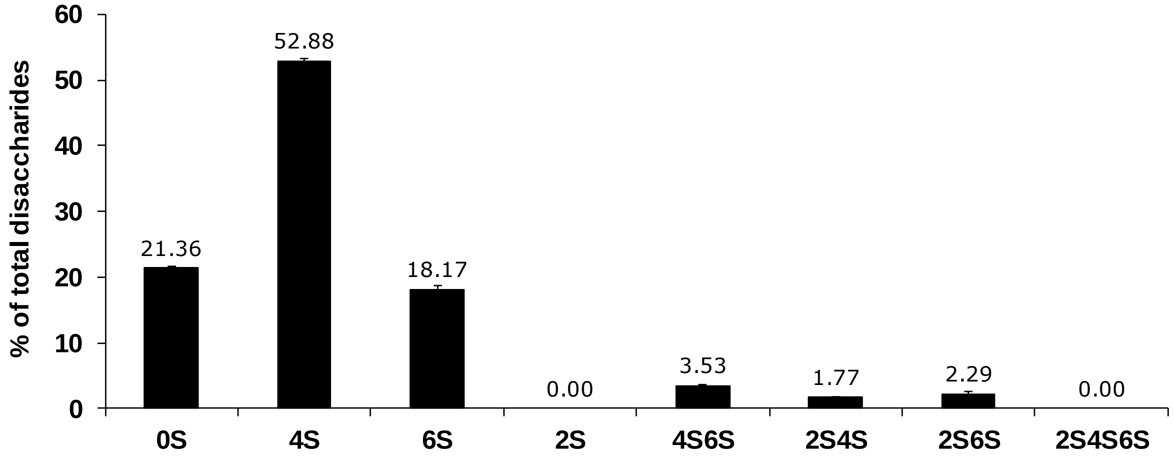

b)

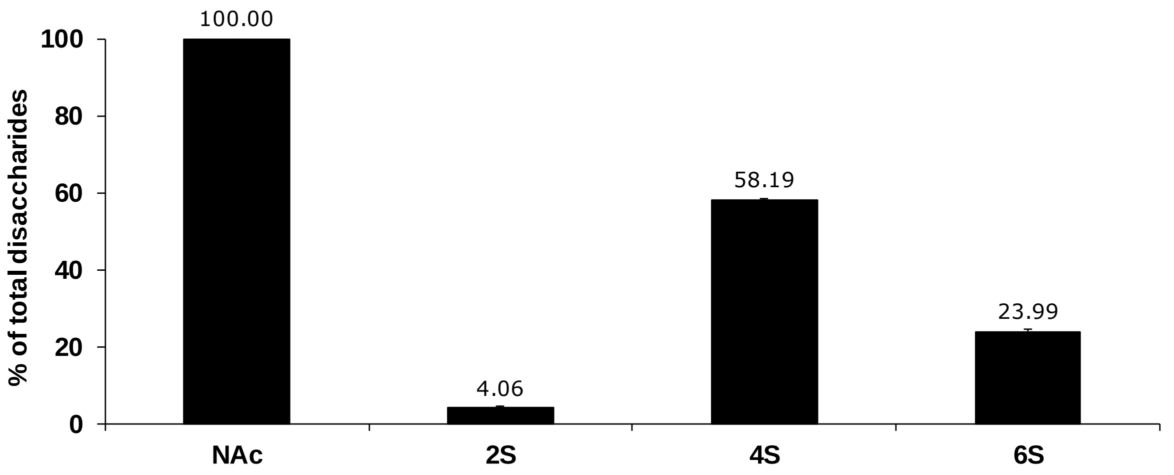

Figure S9. Disaccharide analysis of the CS/DS GAG chain attached to HSulf-2. CS/DS disaccharide composition was determined by RPIP HPLC. a) The relative amount of the different disaccharides. 0S, ΔUA-GalcNAc; 4S, ΔUA-GalcNAc(4S); 6S, ΔUA-GalcNAc(6S); 2S, ΔUA(2S)-GalcNAc; 4S6S, ΔUA-GalcNAc(4S,6S); 2S4S, b) Percentage of disaccharides with different modifications calculated from the data in a).

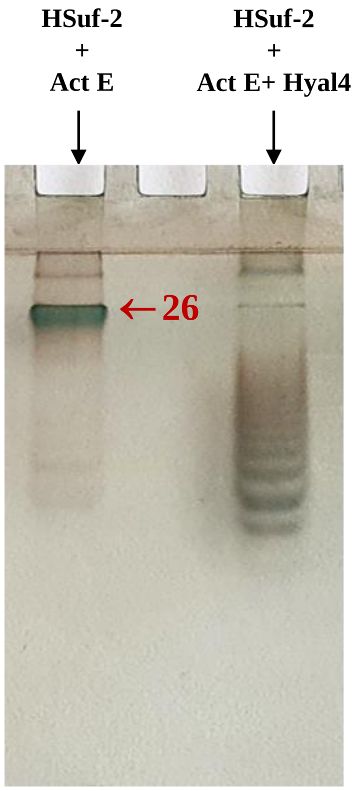

Figure S10. C-PAGE analysis of the depolymerization of the CS/DS linked to HSulf-2 by hyaluronidase. CS/DS chain released from HSulf-2 by Actinase E (1), and subsequent depolymerization by hyaluronidase-4 (2). 27% polyacrylamide gel, Alcian blue/silver nitrate staining.

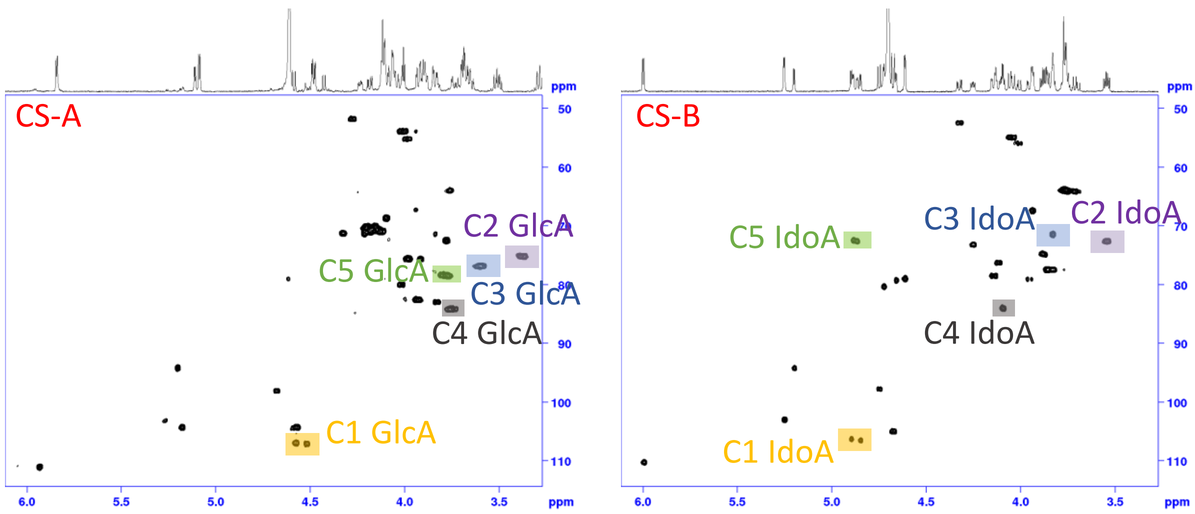

Figure S11. 2D 1H-13C HSQC spectra of CS-A and CS-B tetrasaccharides at 30°C. GlcA and IdoA residue signals of CS-A and CS-B, respectively, are labelled.

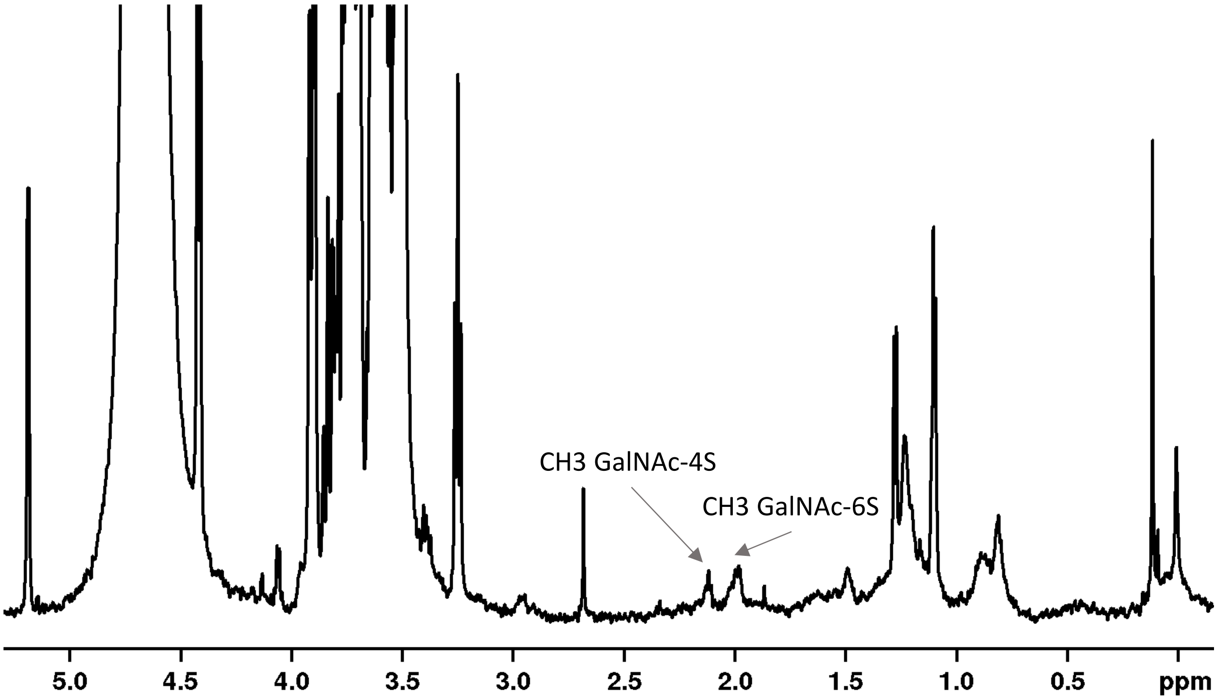

Figure S12. 1D 1H NMR spectrum of the CS/DS oligosaccharides released from HSulf-2 after hyaluronidase depolymerization. Spectrum give clear evidences of the presence of both 4-O-, and 6-O-sulfated GalNAc residues in CS/DS oligosaccharides.

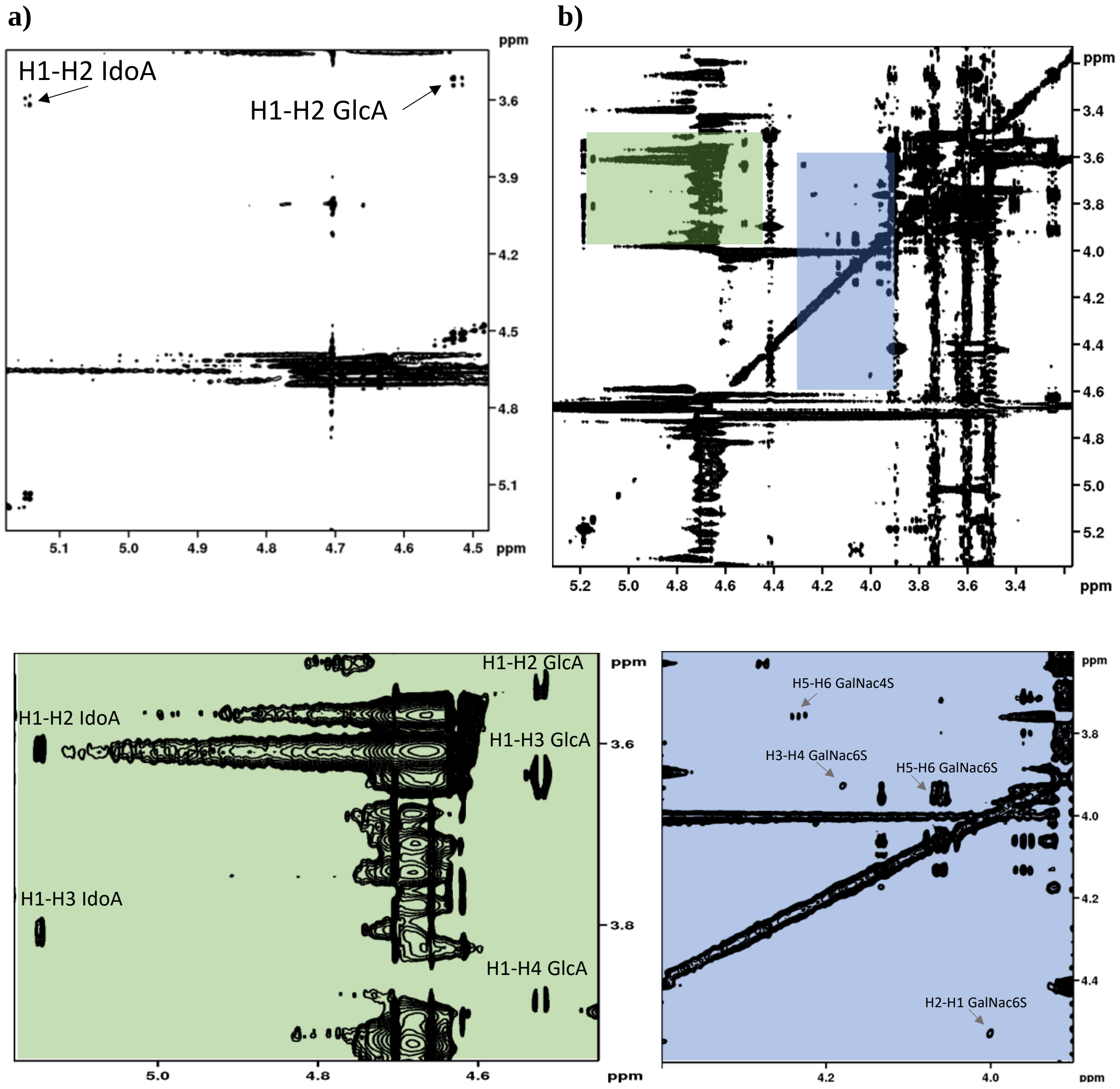

Figure S13. 2D 1H-1H COSY (a) and TOCSY (b) NMR spectrum of the spectrum of the CS/DS oligosaccharides released from HSulf-2 after hyaluronidase depolymerization. Analysis achieved at 30°C. Right inserts in green and blue correspond to zooms on the TOCSY spectrum.

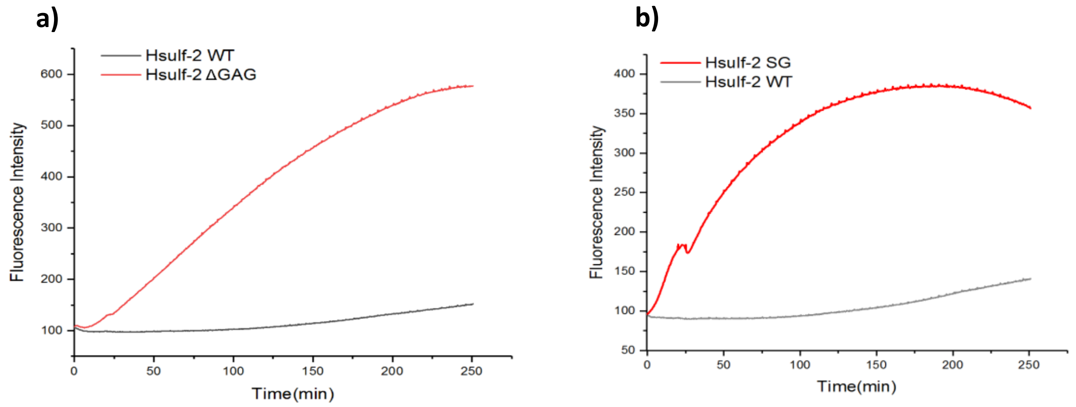

Figure S14. Arylsulfatase activity of HSulf-2 measured on the synthetic substrate 4-MUS. The arylsulfatase of HSulf-2 by comparing (A) the activity of HSulf-2WT (black) to the activity of HSulf-2 ΔGAG (red) whose has been GAG digested by chondroitinase ABC, or (B) of HSulf-2 SG mutant (red) lacking the CS GAG. Arylsulfatase assay at 37°C with 4 mM 4-MUS in 100 µL 50 mM Tris buffer containing 10 mM MgCl$_2$, pH 7.5. The enzyme desulfation of 4-MUS to 4-MU was measured by fluorescence emission at 465 nm.

Table S1. Putative sequences extracted from MALDI-TOF MS analyses of oligosaccharides issued from chondroitinase ABC depolymerization of the HSulf-2-bound GAG. *Sequences in italics represent the least abundant reported in the whole literature*.

dp	Experimental *m/z*	Theoretical *m/z*	Putative sequences
2	582.0	581.9	ΔUA-GalNAc4S6S
	480.0	480.0	ΔUA-GalNAc4S/6S
4	1085.0	1085.0	(ΔUA-GalNAc4S/6S)-(GlcA/IdoA-GalNAc4S6S)<br>(ΔUA-GalNAc4S6S)-(GlcA/IdoA-GalNAc4S/6S)
	983.1	983.1	(ΔUA-GalNAc4S/6S)$_2$<br>*(ΔUA-GalNAc4S6S)-(GlcA/IdoA-GalNAc)*<br>*(ΔUA-GalNAc)-(GlcA/IdoA-GalNAc4S6S)*
	881.1	881.1	*(ΔUA-GalNAc4S/6S)-(GlcA/IdoA-GalNAc)*<br>*(ΔUA-GalNAc)-(GlcA/IdoA-GalNAc4S/6S)*
6	1588.0	1588.0	(ΔUA-GalNAc4S6S)-(GlcA/IdoA-GalNAc4S/6S)$_2$<br>(ΔUA-GalNAc4S/6S)-(GlcA/IdoA-GalNAc4S6S)-(GlcA/IdoA-GalNAc4S/6S)<br>(ΔUA-GalNAc4S/6S)$_2$-(GlcA/IdoA-GalNAc4S6S)<br>*(ΔUA-GalNAc4S6S)$_2$-GlcA/IdoA-GalNAc)*<br>*(ΔUA-GalNAc4S6S)-(GlcA/IdoA-GalNAc)-(GlcA/IdoA-GalNAc4S6S)*<br>*(ΔUA-GalNAc)-(GlcA/IdoA-GalNAc4S6S)$_2$*
	1486.1	1486.1	(ΔUA-GalNAc4S/6S)$_3$<br>*(ΔUA-GalNAc4S6S)-(GlcA/IdoA-GalNAc4S/6S)-(GlcA/IdoA-GalNAc)*<br>*(ΔUA-GalNAc4S6S)-(GlcA/IdoA-GalNAc)-(GlcA/IdoA-GalNAc4S/6S)*<br>*(ΔUA-GalNAc4S/6S)-(GlcA/IdoA-GalNAc4S6S)-(GlcA/IdoA-GalNAc)*<br>*(ΔUA-GalNAc)-(GlcA/IdoA-GalNAc4S6S)-(GlcA/IdoA-GalNAc4S/6S)*<br>*(ΔUA-GalNAc)-(GlcA/IdoA-GalNAc4S/6S)-(GlcA/IdoA-GalNAc4S6S)*<br>*(ΔUA-GalNAc4S/6S)-(GlcA/IdoA-GalNAc)-(GlcA/IdoA-GalNAc4S6S)*

Table S1. *continued*

dp	Experimental *m/z*	Theoretical *m/z*	Putative sequences
8	1989.1	1989.1	(ΔUA-GalNAc4S/6S)$_4$<br>*(ΔUA-GalNAc4S/6S)$_2$-(GlcA/IdoA-GalNAc4S6S)-(GlcA/IdoA-GalNAc)*<br>*(ΔUA-GalNAc4S/6S)$_2$-(GlcA/IdoA-GalNAc)-(GlcA/IdoA-GalNAc4S6S)*<br>*(ΔUA-GalNAc4S/6S)-(GlcA/IdoA-GalNAc4S6S)-(GlcA/IdoA-GalNAc4S/6S)-(GlcA/IdoA-GalNAc)*<br>*(ΔUA-GalNAc4S/6S)-(GlcA/IdoA-GalNAc4S6S)-(GlcA/IdoA-GalNAc)-(GlcA/IdoA-GalNAc4S/6S)*<br>*(ΔUA-GalNAc4S/6S)-(GlcA/IdoA-GalNAc)-(GlcA/IdoA-GalNAc4S6S)-(GlcA/IdoA-GalNAc4S/6S)*<br>*(ΔUA-GalNAc4S/6S)-(GlcA/IdoA-GalNAc)-(GlcA/IdoA-GalNAc4S/6S)-(GlcA/IdoA-GalNAc4S6S)*<br>*(ΔUA-GalNAc4S6S)$_2$-(GlcA/IdoA-GalNAc)$_2$*<br>*(ΔUA-GalNAc4S6S)-(GlcA/IdoA-GalNAc)$_2$-(GlcA/IdoA-GalNAc4S6S)*<br>*(ΔUA-GalNAc4S6S)-(GlcA/IdoA-GalNAc)-(GlcA/IdoA-GalNAc4S6S)-(GlcA/IdoA-GalNAc)*<br>*(ΔUA-GalNAc)-(GlcA/IdoA-GalNAc4S6S)$_2$-(GlcA/IdoA-GalNAc)*<br>*(ΔUA-GalNAc)-(GlcA/IdoA-GalNAc4S6S)-(GlcA/IdoA-GalNAc)-(GlcA/IdoA-GalNAc4S6S)*<br>*(ΔUA-GalNAc)$_2$-(GlcA/IdoA-GalNAc4S6S)$_2$*<br>*(ΔUA-GalNAc4S6S)-(GlcA/IdoA-GalNAc4S/6S)$_2$-(GlcA/IdoA-GalNAc)*<br>*(ΔUA-GalNAc4S6S)-(GlcA/IdoA-GalNAc4S/6S)-(GlcA/IdoA-GalNAc)-(GlcA/IdoA-GalNAc4S/6S)*<br>*(ΔUA-GalNAc4S6S)-(GlcA/IdoA-GalNAc)-(GlcA/IdoA-GalNAc4S/6S)$_2$*<br>*(ΔUA-GalNAc)-(GlcA/IdoA-GalNAc4S/6S)$_2$-(GlcA/IdoA-GalN4S6S)*<br>*(ΔUA-GalNAc)-(GlcA/IdoA-GalNAc4S/6S)-(GlcA/IdoA-GalN4S6S)-(GlcA/IdoA-GalNAc4S/6S)*<br>*(ΔUA-GalNAc)-(GlcA/IdoA-GalNAc4S6S)-(GlcA/IdoA-GalN4S/6S)$_2$*

Table S2. Identification of the five *N*-glycans located within glycopeptides at Asn88, Asn125, Asn174, and Asn217 on the long chain and at Asn537 on the short chain of HSulf-2.

T (min)	*Glycopeptides*				*N-glycan*	
	Exp. m/z (4+)	*Calc. m/z (4+)*	*Δm (ppm)*	*Ascribed sequence (Asn residue number in red)*	*Deducted mass*	*Composition*
12.60	*1202.2285*	*1202.2282*	*0.2*	*YVHNHNTYTNNEN^{88}CSSPSWQAQHESR*	*1702.5842*	*HexNAc(2)Hex(8)*
13.03	*998.7252*	*998.7243*	*0.9*	*VYHVGLGDAAQPRN^{537}LTKR*	*1996.7869*	*HexNAc(6)Hex(3)Fuc(2)*
20.57	*1 274.3281*	*1274.3297*	*1.3*	*LFPN^{217}ASQHITPSYNYAPNPDKHWIMR*	*1996.7810*	*HexNAc(6)Hex(3)Fuc(2)*
25.18	*1039.2092*	*1039.2087*	*0.5*	*EKHGSDYSKDYLTDLITN^{174}DSVSFFR*	*1216.4248*	*HexNAc(2)Hex(5)*
28.68	*1120.7775*	*1120.7753*	*2.0*	*TAFFGKYLNEYN^{125}GSYVPPGWKEWVGLLK*	*1216.4316*	*HexNAc(2)Hex(5)*

Table S3. List of the most abundant glycopeptides containing Asn residue issued from trypsin and PNGase F treatment and detected after CID/ETD analysis. Each site of glycosylation was confirmed by the detection of deamidation introduced by PNGase F treatment (+0.984 mass increment on the MS/MS spectra of de-glycosylated peptides). Exact composition and relative abondance of each precursor were estimated using Byologic Protein Metric software (see experimental section) and manually confirmed.

**Identified peptide sequence**	**Chain**
SSILTGKYVHNHNTYTNNE**N**^{88}CSSPSWQAQHESR	
YVHNHNTYTNNE**N**^{88}CSSPSWQAQHESR	
TAFFGKYLNEY**N**^{125}GSYVPPGWKEWVGLLK	
YLNEY**N**^{125}GSYVPPGWK	
YLNEY**N**^{125}NGSYVPPGWKEWVGLLK	
YLNEY**N**^{125}GSYVPPGWKEWVGLLKNSR	Short
DYLTDLIT**N**^{174}DSVSFFR	
EKHGSDYSKDYLTDLIT**N**^{174}DSVSFFR	
HGSDYSKDYLTDLIT**N**^{174}DSVSFFR	
LFP**N**^{217}NASQHITPSYNYAPNPDKHWIMR	
P**N**^{217}ASQHITPSYNYAPNPDKHWIMR	
GLGDAAQPR**N**^{537}LTKR	
GRVYHVGLGDAAQPR**N**^{537}LTK	
HVGLGDAAQPR**N**^{537}LTK	
HVGLGDAAQPR**N**^{537}LTKR	Long
SVAIEVDGRVYHVGLGDAAQPR**N**^{537}LTK	
VYHVGLGDAAQPR**N**^{537}LTK	
VYHVGLGDAAQPR**N**^{537}LTKR	

a)

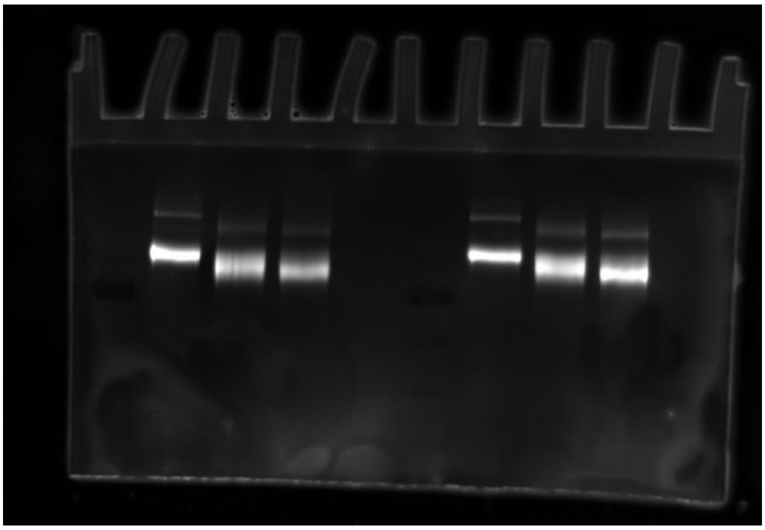

b)

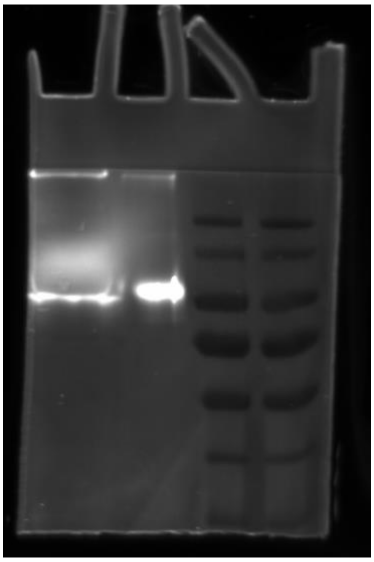

Figure S15. Uncropped, full-length SDS-PAGE gels corresponding to (a) the SDS-PAGE analysis shown in figure 6A and (b) the SDS-PAGE analysis shown in figure 6C., detection with the SNAP-Vista Green.
